# Hybrid macrocycle formation and spiro annulation on *cis-syn-cis-*tricyclo[6.3.0.0^2,6^]undeca-3,11-dione and its congeners via ring-closing metathesis

**DOI:** 10.3762/bjoc.11.126

**Published:** 2015-07-06

**Authors:** Sambasivarao Kotha, Ajay Kumar Chinnam, Rashid Ali

**Affiliations:** 1 Department of Chemistry, Indian Institute of Technology-Bombay, Powai, Mumbai-400 076, India, Fax: 022-2572 7152

**Keywords:** aza-polyquiananes, Fischer indolization, macrocycles, ring-closing metathesis, spiropolyquinanes

## Abstract

We have developed a simple methodology to transform *cis-syn-cis-*triquinane derivative **2** into the diindole based macrocycle **6** involving Fischer indolization and ring-closing metathesis (RCM). Various spiro-polyquinane derivatives have been assembled via RCM as a key step.

## Introduction

**Figure 1:**
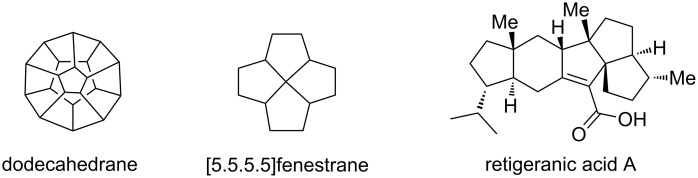
Natural and non-natural products containing quinane systems.

Design and synthesis of polyquinanes is an active area of research during the last three decades [1–10]. Various theoretically interesting as well as biologically active molecules such as dodecahedrane, [5.5.5.5]fenestrane and retigeranic acid A contain the quinane framework in their structures (Figure 1). A variety of quinane-based natural products isolated from terrestrial, microbial and marine sources have stimulated the growth of polyquinane chemistry. In this context, there is a continuous demand for the development of new methodologies to assemble cyclopentanoids (or quinanes) [11–21]. Several approaches are available for the synthesis of carbocyclic quinanes, however, only a limited number of methods is available for oxa- [22–25] and aza-polyquinanes [26–28]. The indole unit is present in a variety of plant alkaloids (e.g., reserpine, strychnine, physostigmine) and several important drugs contain indole as a key component [29–32]. Therefore, we are interested in designing new strategies to hybrid molecules containing both quinane and indole ring systems. On several occasions, the spirocyclic moiety seems to be a recurring motif in bioactive molecules. Consequently, assembling architecturally complex spirocycles is of great relevance to the diversity-oriented synthesis of biologically active spirocycles. In this context, new synthetic methods to generate multiple spirocenters in a simple manner remain a challenging task. Although, a variety of strategies have been investigated, a limited number of general methods are available [33–46] for the generation of multiple spirocenters in a single step [43]. To expand the chemical space of aza-polyquinanes we conceived a new strategy based on Fischer indolization and ring-closing metathesis as the key steps.

**Figure 2:**
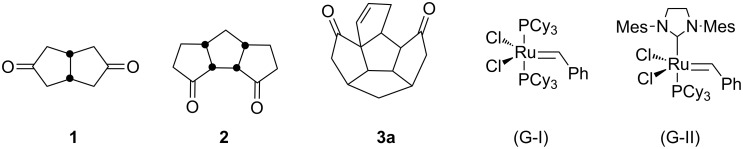
Quinane building blocks (**1**–**3**) and metathetic catalyst used in our strategy.

To develop a simple synthetic methodology to aza-polycycles and spiropolycycles from readily available starting materials [47–52], bicyclic, tricyclic and pentacyclic diones (**1**–**3**) were identified as useful building blocks (Figure 2). The selection of these diones is based on their easy accessibility and also the symmetry involved with them. For example, with diones **1** and **2** one can apply a two-directional synthesis [53] to increase the brevity [54] of the overall synthesis. Earlier, we have shown that Weiss–Cook dione **1** [49–51] is a useful substrate for double Fischer indolization with a low melting mixture of L-(+)-tartaric acid and *N*,*N*′-dimethylurea (L-(+)-TA:DMU) [55] at 70 °C to generate an unusual *C*_s_-symmetric diindole derivative along with the known *C*_2_-symmetric diindole [56]. Also, based on Fischer indolization and ring-closing metathesis (RCM), we have developed a new strategy to indole-based propellane derivatives [57].

**Scheme 1:**
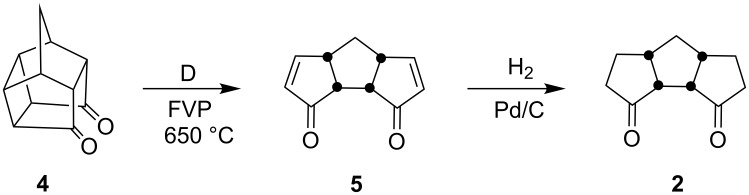
Synthesis of tricyclic diones **5** and **2**.

Here, the tricyclic dione **2** required was prepared starting with the Cookson’s dione **4** in two steps involving flash vacuum pyrolysis (FVP) and hydrogenation steps (Scheme 1). A variety of synthetic transformations involving tricyclic diones **5** and **2** were reported in the literature [47].

**Figure 3:**
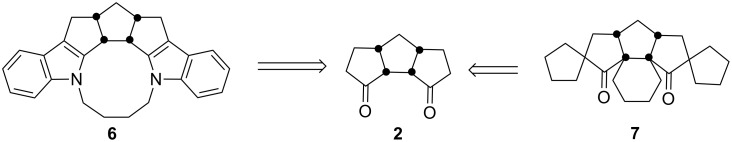
Retrosynthetic approach to aza-polyquinane **6** and spiro-polyquinane **7**.

To expand the utility of building block **2** in organic synthesis, we conceived a simple retrosynthetic approach to macrocylic aza-polyquinane **6** and spiro-polyquinane derivative **7** (Figure 3). The key steps involved here are: double Fischer indolization and RCM. To install the alkane chain connecting the two nitrogen atoms, we plan to use alkylation with allylbromide followed by RCM and hydrogenation protocols. It is known that a mono-indole derivative was obtained via Fischer indolization starting with dione **2** and two equivalents of phenylhydrazine hydrochloride, but the diindole derivative **8** [58] was not obtained under these conditions. Our experience with Fischer indolization of **1** using the low melting mixture protocol gave unusual results as compared with conventional Fischer indolization conditions. Therefore, the reactivity of **2** under conditions of the low melting mixture is worthy of systematic investigation. Here, we are pleased to report our successful results in generating the diindole derivative **8** by utilizing a low melting mixture of L-(+)-TA:DMU and its subsequent utility in assembling the macrocyclic system **6** via RCM. During this venture, we also found that the tricyclic dione **2** is a useful substrate for the synthesis of spiro-polyquinane derivative **7** via a six fold allylation followed by a three-fold RCM and a hydrogenation sequence.

## Results and Discussion

**Scheme 2:**
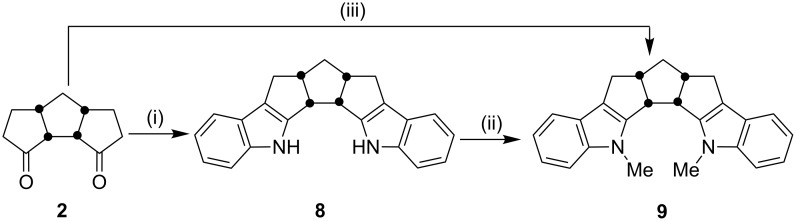
Synthesis of the diindole derivative **9** Reagents and conditions: (i) TA:DMU, PhNHNH_3_Cl, 70 °C, 6 h, 62%; (ii) NaH, MeI, DMF, rt, 24 h, 87%; (iii) TA:DMU, 70 °C, PhNMeNH_2_, 6 h, 76%.

To realize the strategy shown in Figure 3, the tricyclic dione **2** was subjected to a two-fold Fischer indolization in the presence of two equivalents of phenylhydrazine hydrochloride with the aid of a low melting mixture of L-(+)-TA:DMU to generate the diindole derivative **8** (62%, Scheme 2). The structure of the diindole **8** has been established on the basis of ^1^H NMR and ^13^C NMR spectral data. The presence of 12 signals in the ^13^C NMR spectrum clearly indicated that the *C*_s_-symmetry is present in molecule **8**. Later, the diindole derivative was treated with methyl iodide in the presence of NaH/DMF at room temperature to deliver the dimethyl derivative **9**. Again, the *C*_s_-symmetry present in **9** is evidenced by the appearance of 13 signals in the ^13^C NMR spectrum. Alternatively, the diindole derivative **9** has been generated in a single step by reacting the dione **2** with *N*-methyl-*N*-phenylhydrazine under conditions using the described low melting mixture.

**Scheme 3:**
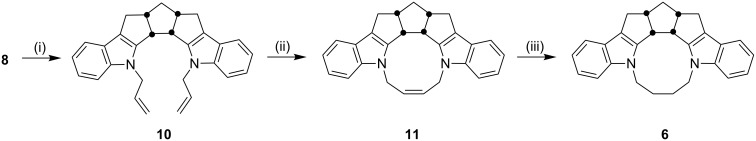
Synthesis of the macrocyclic aza-polyquinane derivative **6**. Reagents and conditions: (i) NaH, allyl bromide, DMF, rt, 24 h, 65%; (ii) G-II, CH_2_Cl_2_, rt, 12 h, 84%; (iii) Pd/C, H_2_, EtOAc, rt, 18 h, 95%.

Next, the *N*-allylation of the diindole derivative **8** with allyl bromide in the presence of NaH/DMF gave diallyl derivative **10**, which was subjected to the RCM sequence in the presence of Grubbs’ 2^nd^ generation catalyst to produce the cyclized compound **11** (84%). Subsequently, the macrocyclic diindole derivative **11** was hydrogenated in the presence of H_2_/Pd/C to afford the saturated compound **6** (Scheme 3).

**Scheme 4:**
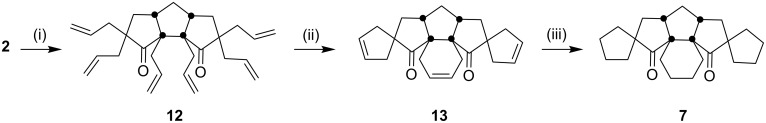
Synthesis of the spiro-polyquinane **7**. Reagents and conditions: (i) NaH, allyl bromide, THF, rt, 24 h, 59%; (ii) G-I, CH_2_Cl_2_, rt, 15 h, 80%; (iii) Pd/C, H_2_, EtOAc, rt, 24 h, 90%.

To assemble the intricate spiro-polyquinane **7** via RCM as a key step [59–62], we started with the triquinane derivative **2**. To this end, the *cis-syn-cis*-triquinane dione **2** was treated with an excess amount of allyl bromide in the presence of NaH to generate the hexaallyl derivative **12** in 59% yield. Later, it was subjected to RCM with Grubbs’ 1^st^ generation catalyst to deliver the three-fold RCM product **13** in 80% yield. Furthermore, treatment of the hexacyclic dione **13** with Pd/C in EtOAc under hydrogen atmosphere (1 atm) gave the saturated spiro-polyquinane **7** in 90% yield (Scheme 4). Very few examples are known in the literature where multiple RCM was performed in a single operation to generate the molecules of medium molecular weight [63]. The present example involving the generation of triple spirocyclic compound **7** is unique and demonstrates the power and scope of the RCM approach. It is worth mentioning that previous attempts to functionalize **2** were unsuccessful [47].

**Scheme 5:**
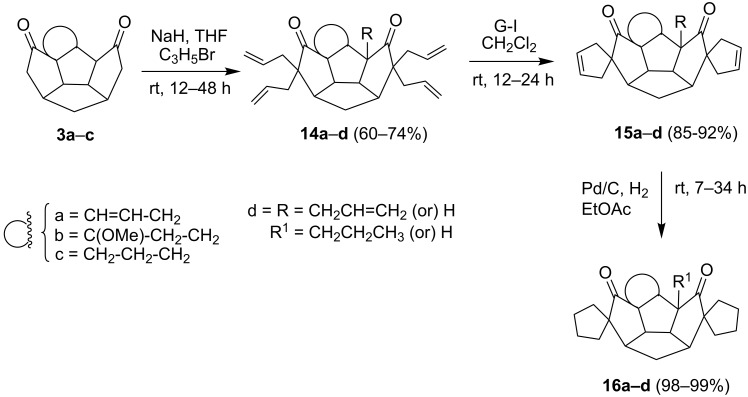
General strategy to bis-spirocycles via RCM.

To generalize the spiroannulation sequence, allylation of pentacyclic diones **3a–c** [48] gave the tetraallyl diones **14a–c** in respectable yields. Next, treatment of these allylated derivatives **14a–c** with G-I catalyst gave the double RCM products **15a**, **15b** and **15c** in 92%, 92% and 91% yields, respectively (Scheme 5 and Table 1). Later, these double RCM products were subjected to the hydrogenation protocol in the presence of H_2_/Pd/C to deliver the saturated bis-spiro-polyquinane derivatives **16a**, **16b** and **16a** in an excellent yield (Table 1). Similarly, the dione **3a** in the presence of an excess amount of NaH and allyl bromide gave the pentaallyl dione **14d** in 67% yield (Table 1). Next, the pentaallyl derivative **14d** was treated with G-I catalyst to produce the bis-spiro-polyquinane **15d**. ^1^H NMR and ^13^C NMR spectral data clearly indicated the presence of intact allyl residue along with the unsaturated double bonds. The bis-spiro-polyquinane **15d** was then subjected to hydrogenation sequence to deliver the saturated bis-spiro-polyquinane **16d** in good yield (Table 1).

**Table 1:** List of bis-spirocycles assembled by RCM.

Allylation product (%)	Time	RCM product (%)	Time	Hydrogenation product (%)	Time

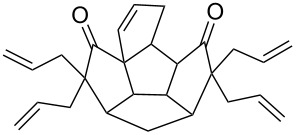 **14a** (74%)	12 h	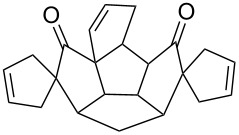 **15a** (92%)	12 h	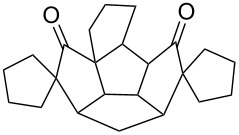 **16a** (98%)	7 h
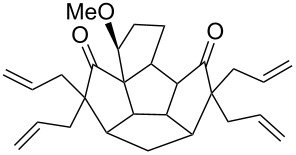 **14b** (60%)	14 h	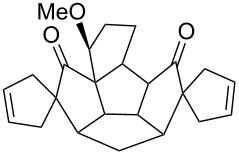 **15b** (92%)	10 h	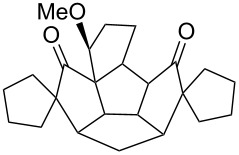 **16b** (99%)	7 h
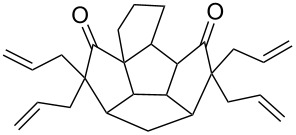 **14c** (70%)	12 h	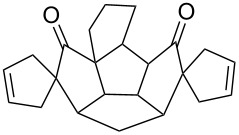 **15c** (91%)	12 h	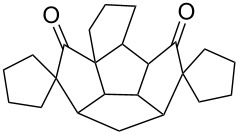 **16a** (99%)	7 h
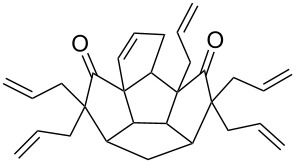 **14d** (67%)	48 h	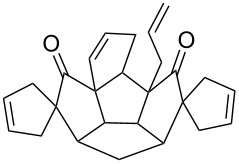 **15d** (85%)	24 h	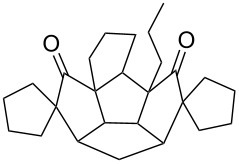 **16d** (97%)	12 h

## Conclusion

In summary, we have developed a protocol for the synthesis of a diindole-based hybrid macrocycle through Fischer indolization of the triquinane **2** followed by bis-*N*-allylation and RCM. The allylation-RCM sequence has also been extended to construct structurally intricate spiro-polyquinanes.

## Supporting Information

File 1 Experimental and analytical data.

File 2 NMR spectra.
