# Supplementary material for: Hybrid macrocycle formation and spiro annulation on cis-syn-cis-tricyclo[6.3.0.02,6]undeca-3,11-dione and its congeners via ring-closing metathesis
Source: Beilstein J Org Chem. 2015 Jul 6;11:1123–8. doi: 10.3762/bjoc.11.126 (PMC4505178; doi:10.3762/bjoc.11.126)
Supplement: File 1 — Experimental and analytical data. [file Beilstein_J_Org_Chem-11-1123-s001.pdf]

Supporting Information File 1

for

Hybrid macrocycle formation and spiro annulation on *cis*-*syn*-*cis*-tricyclo[6.3.0.0^{2,6}]undeca-3,11-dione and its congeners via ring-closing metathesis

Sambasivarao Kotha*, Ajay Kumar Chinnam and Rashid Ali

Address: Department of Chemistry, Indian Institute of Technology-Bombay, Powai, India, Fax: 022-2572 7152

Email: Sambasivarao Kotha - srk@chem.iitb.ac.in

*Corresponding author

Experimental and analytical data

General

Melting points were recorded on a Buchi apparatus. Infrared (IR) spectra were recorded on a Nicolet Impact-400 FT IR spectrometer in KBr/$CHCl_3$. ^{1}H NMR (400 MHz), ^{13}C NMR (100 MHz) spectral data were obtained at rt on Bruker (AVANCE IIITM) 500 MHz and Bruker (AVANCE IIITM) 400 MHz spectrometers with tetramethylsilane (TMS) as an internal standard and in $CDCl_3$ solution. Coupling constants (*J* values) are given in Hertz (Hz). High-resolution mass spectrometric (HRMS) measurements were carried out using Bruker (Maxis Impact) or Micromass Q-ToF spectrometers. Analytical thin layer chromatography (TLC) was performed on (10 × 5 cm) glass plates coated with Acme's silica gel GF 254 (containing 13% calcium sulfate as a binder). Chromatography was performed using Acme's silica gel (100-200 mesh) using double spray bellows for application of pressure and the column is eluted with ethyl acetate–petroleum ether mixture. All of the organic solvents used in this study were dried over appropriate drying agents and distilled prior to use.

General procedure for the preparation of diindole derivatives.

In a typical experiment, 1.5 g of an L-(+)-tartaric acid/*N*,*N′*-dimethylurea (30:70) mixture was heated to 70 °C to obtain a clear melt. To this melt, 2 mmol of *N*-methyl-*N*-phenylhydrazine and 1 mmol of diketone were added at 70 °C. After termination of the reaction (TLC monitoring by mini work up), the reaction mixture was quenched with water while it was still hot. The reaction mixture was cooled to rt and the solid was filtered through a sintered glass funnel and washed with water (2 × 5 mL). The solvent was evaporated and the crude product was purified by silica gel column chromatography with an appropriate mixture of EtOAc and petroleum ether [1,2].

Synthesis of compound 8

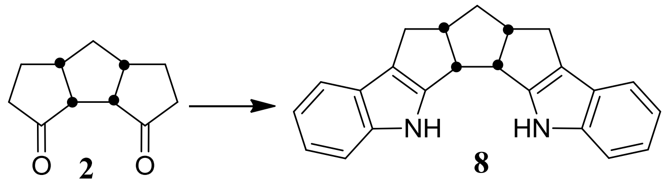

2 **8**

The tricyclic dione **2** (100 mg, 0.56 mmol), was reacted with phenylhydrazine hydrochloride (177 mg, 1.23 mmol) by following the general procedure. After termination of the reaction (TLC monitoring), the reaction mixture was worked up according to the general procedure. The crude product obtained was purified by column chromatography on silica gel

(EtOAc/petroleum ether, 10:90) to provide the diindole derivative **8** (115 mg, 62%) as a colourless solid [2].

Mp 189-191 °C; R_f = 0.41 (Silica gel, 10% EtOAc/petroleum ether); ^{1}H NMR (400 MHz, $CDCl_3$) δ = 1.52-1.60 (m, 1H), 2.45-2.54 (m, 3H), 2.89-2.95 (q, J = 7.9 Hz, 2H), 3.46-3.51 (m, 2H), 3.87-3.89 (m, 2H), 6.99-7.06 (m, 4H), 7.18-7.20 (m, 2H), 7.31-7.33 (m, 2H), 7.58 (bs, 2H); ^{13}C NMR (100 MHz, $CDCl_3$) δ = 31.38, 41.43, 48.22, 51.71, 111.82, 118.91, 119.88, 120.27, 120.94, 125.56, 141.58, 144.02; accurate mass (ESI, Q-ToF) *m/z*: calculated for $C_{23}H_{20}KN_2$ $[M+K]^+$: 363.1258; found: 363.1254.

Synthesis of compound 9

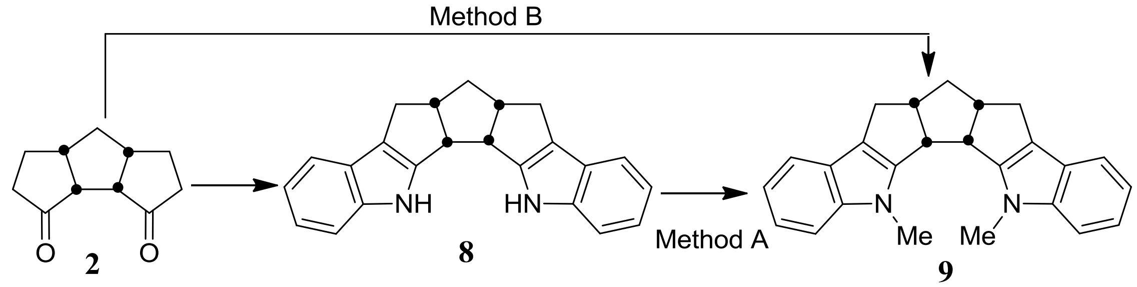

2 **8** **9**

Method A

To a suspension of NaH (8.2 mg, 0.34 mmol) in DMF (5 mL), the diindole **8** (50 mg, 0.15 mmol) was added at rt under nitrogen. Then, the reaction mixture stirred at rt for 15 min. Next, methyl iodide (0.03 mL, 0.34 mmol) was added in a dropwise manner, and then the stirring was continued for 24 h at rt. After termination of the reaction (TLC monitoring), the reaction mixture was diluted with ethyl acetate (10 mL), and the organic layer was washed with water, brine and dried over Na_2SO_4. The solvent was removed under reduced pressure. The crude product obtained was purified by silica gel column chromatography (5% EtOAc/petroleum ether) to yield compound **9** (47 mg, 87%) [3].

Method B

The tricyclic dione **2** (50 mg, 0.28 mmol) was reacted with *N*-methyl-*N*-phenylhydrazine (76 mg, 0.61 mmol) following the general procedure. After termination of the reaction (TLC monitoring), the reaction mixture was worked up according to the general procedure. The crude product obtained was purified by column chromatography on silica gel (EtOAc/petroleum ether, 10:90) to provide the diindole derivative **9** (75 mg, 76%) as a colourless solid [2].

Mp 206-208 °C; R_f = 0.39 (Silica gel, 10% EtOAc-petroleum ether); ^{1}H NMR (400 MHz, $CDCl_3$) δ = 1.74-1.80 (m, 1H), 2.43-2.55 (m, 3H), 2.85-2.88 (m, 2H), 3.24 (s, 6H), 3.61-3.64 (m, 2H), 3.74-3.78 (m, 2H), 7.00-7.08 (m, 6H), 7.33 (d, J = 7.7 Hz, 2H); ^{13}C NMR (100 MHz, $CDCl_3$) δ = 30.70, 32.48, 38.39, 48.95, 54.48, 109.60, 118.82, 119.10, 120.31, 124.65, 141.29, 146.09; accurate mass (ESI, Q-ToF) *m/z*: calculated for $C_{25}H_{24}N_2Na$ $[M+Na]^+$: 375.1832; found: 375.1835.

Synthesis of compound 10

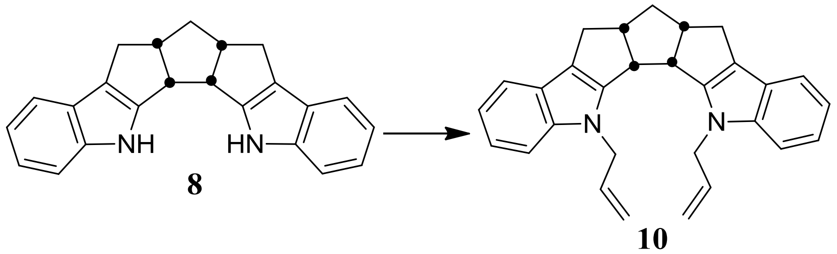

To a suspension of NaH (16.3 mg, 0.70 mmol) in DMF (10 mL), the diindole **8** (100 mg, 0.3 mmol) was added at rt under nitrogen. Then, the reaction mixture stirred at rt for 15 min. Allyl bromide (0.02 mL, 0.3 mmol) was added in a dropwise manner, and then the stirring was continued at rt for 24 h. After termination of the reaction (TLC monitoring), the reaction mixture was diluted with ethyl acetate (10 mL), and the organic layer was washed with water, brine and dried over Na_2SO_4. The solvent was removed under reduced pressure. The crude product obtained was purified by silica gel column chromatography (5% EtOAc/petroleum ether) to yield compound **10** (81 mg, 65%) as a yellow thick liquid [3].

R_f = 0.42 (Silica gel, 5% EtOAc-petroleum ether); ^{1}H NMR (400 MHz, $CDCl_3$): δ = 1.77-1.83 (m, 1H), 2.44-2.56 (m, 3H), 2.86-2.90 (m, 2H), 3.59-3.63 (m, 2H), 3.73-3.74 (m, 2H), 3.96-4.01 (m, 2H), 4.35-4.39 (m, 2H), 4.74-4.78 (m, 2H), 4.96-4.98 (m, 2H), 5.62-5.67 (m, 2H), 7.00-7.11 (m, 6H), 7.33-7.35 (m, 2H); ^{13}C NMR (100 MHz, $CDCl_3$): δ = 32.20, 38.29, 47.03, 49.37, 54.56, 110.37, 115.98, 118.89, 119.32, 120.05, 120.50, 125.13, 134.15, 141.02, 145.72; accurate mass (ESI, Q-ToF) *m/z*: calculated for $C_{29}H_{28}N_2Na$ $[M+Na]^+$: 427.2145; found: 427.2150.

Synthesis of compound 11

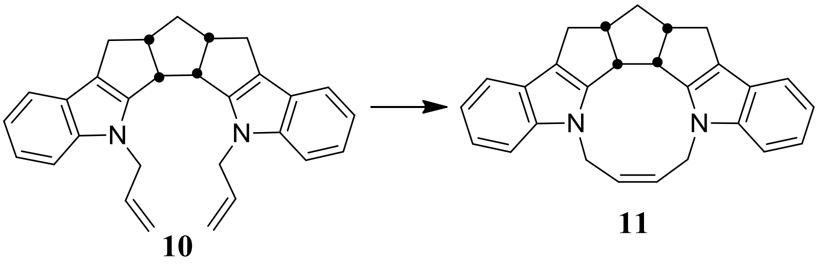

A solution of diallyldiindole **10** (65 mg, 0.16 mmol) in dry CH_2Cl_2 (15 mL) was degassed with N_2 for 10 min. Then, G-II (7.5 mol %) was added stirring was continued for 24 h at rt. After termination of the reaction (TLC monitoring), the solvent was removed at reduced pressure and the crude product was purified by silica gel column chromatography (5% EtOAc/petroleum ether) gave the RCM compound **11** (51 mg, 84%) as a white solid [3].

Mp at 235 ^{o}C decomposed; R_f = 0.40 (Silica gel, 5% EtOAc/petroleum ether); ^{1}H NMR (400 MHz, $CDCl_3$): δ = 1.74-1.80 (m, 1H), 2.45-2.58 (m, 3H), 2.89-2.94 (m, 2H), 3.54-3.63 (m, 2H), 3.83-3.95 (m, 4H), 4.37-4.40 (d, J = 13.9 Hz, 2H), 5.73-5.75 (t, J = 3.6 Hz, 2H), 6.98-7.09 (m, 4H), 7.14-7.16 (m, 2H), 7.33-7.35 (m, 2H); ^{13}C NMR (100 MHz, $CDCl_3$): δ = 32.20, 39.80, 41.41, 49.50, 53.31, 109.31, 118.97, 119.17, 119.86, 120.51, 124.50, 127.55, 140.57, 145.39; accurate mass (ESI, Q-ToF) *m/z*: calculated for $C_{27}H_{24}N_2Na$ $[M+Na]^+$: 399.1832; found: 399.1835

Synthesis of compound 6

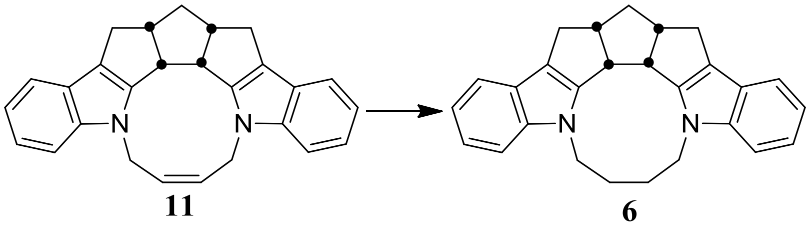

To a solution of compound **11** (50 mg, 0.13 mmol) in dry EtOAc (10 mL), 10 mol% Pd/C (15.4 mg, 0.13 mmol), was added and the reaction mixture was stirred at rt under H_2 pressure (1 atm) for 32 h. After termination of the reaction (TLC monitoring), the reaction mixture was filtered through a Celite-pad and washed with ethyl acetate (20 mL). Evaporation of the solvent at reduced pressure gave the crude product. Further purification by silica-gel column chromatography (5% EtOAc/petroleum ether) gave the hydrogenated product **6** (48 mg, 95%) as a white solid [3].

Mp 245-247 ^{o}C; R_f = 0.42 (Silica gel, 5% EtOAc-petroleum ether); ^{1}H NMR (500 MHz, $CDCl_3$): δ = 1.83-1.88 (m, 1H), 1.96-2.02 (m, 4H), 2.42-2.49 (m, 1H), 2.66 (d, J = 4.4 Hz, 1H), 2.69 (d, J = 4.5 Hz, 1H), 2.92-2.99 (m, 2H), 3.42-3.45 (m, 2H), 3.68-3.71 (m, 2H), 3.97-4.02 (m, 4H), 6.99-7.04 (m, 2H), 7.06-7.11 (m, 2H), 7.15-7.18 (m, 2H), 7.34 (d, J = 7.7 Hz, 2H); ^{13}C NMR (126 MHz, $CDCl_3$) δ = 26.57, 31.89, 39.11, 43.41, 49.71, 54.25, 109.81, 118.94, 119.07, 119.72, 120.45, 124.51, 140.32, 145.42; accurate mass (ESI, Q-ToF) *m/z*: calculated for $C_{27}H_{27}N_2$ $[M+H]^+$: 379.2168 found: 379.2165.

General procedure for the allylation of 2 and 3a–d

To a suspension of NaH (6 equivalents) in THF (20 mL), added the dione **2** (or **3a–c)** at rt under nitrogen and the reaction mixture was stirred at rt for 10 min. Later, allyl bromide was added to the reaction mixture in a dropwise manner, and stirring was continued overnight at rt. After termination of the reaction (TLC monitoring), the reaction mixture was diluted with ethyl acetate (10 mL), washed with water, brine and dried over Na_2SO_4, concentrated at reduced pressure. The crude product obtained was purified by silica gel column chromatography with appropriate mixture of EtOAc-petroleum ether to yield the allylated product **12** (or **14a–d**) [4].

General procedure of the ring-closing metathesis of 12 and 14a–d

A solution of compound **12** (or **14a–d**) in dry CH_2Cl_2 (20 mL) was degassed with N_2 for 10 min, then G-I (10 mol%) was added and stirring was continued for 12–24 h at rt. After termination of the reaction (TLC monitoring), the solvent was removed under reduced pressure and the crude product was purified by silica gel column chromatography with an appropriate mixture of EtOAc/petroleum ether to deliver the desired RCM product **13** (or **15a-d**) [4].

General procedure for the hydrogenation of 13 and 15a–d

The solution of compounds **13** (or **15a–d**) in EtOAc (10 mL), 10 mol % Pd/C (1 mmol) was added and the reaction mixture was stirred at rt under H_2 pressure (1 atm) for 6–24 h. After termination of the reaction (TLC monitoring), the reaction mixture was filtered through using a Celite-pad and the solvent was removed under reduced pressure. The crude product was purified by silica-gel column chromatography by using an appropriate mixture of EtOAc/petroleum ether to yield the desired hydrogenated product **7** (or **16a–d**) [4].

Synthesis of compound 12

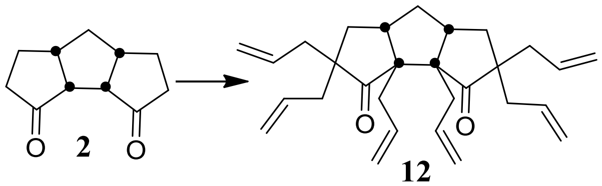

Thick colorless liquid, 59% yield (141.0 mg, starting with 100.0 mg of tricyclic dione **2**)

R_f = 0.74 (silica gel, 10% EtOAc-petroleum ether): ^{1}H NMR (400 MHz, $CDCl_3$) δ = 1.29-1.48 (m, 3H), 1.60-1.73 (m, 2H), 1.78-1.91 (m, 2H), 2.01-2.78 (m, 13H), 4.89-5.30 (m, 12H), 5.70-5.97 (m, 6H); ^{13}C NMR (100 MHz, $CDCl_3$) δ = 37.21, 38.05, 38.47, 40.38, 45.13, 52.45, 67.44, 118.25, 118.28, 118.58, 133.61, 134.13, 134.31, 220.71; IR υ_{max} = 1638, 1729, 2851, 2927 cm^{-1}; accurate mass (ESI, Q-ToF) *m/z*: calculated for $C_{29}H_{38}NaO_2$ $[M+Na]^+$ 441.2764 found: 441.2765.

Synthesis of compound 13

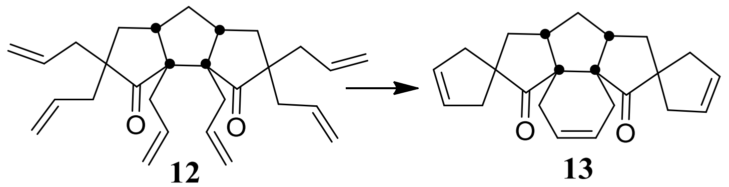

Colorless semi-solid, 80% yield (45.0 mg, starting with 70.0 mg of hexa-allyl tricyclic dione **12**)

R_f = 0. 70 (silica gel, 10% EtOAc-petroleum ether): ^{1}H NMR (400 MHz, $CDCl_3$) δ = 1.26-1.33 (m, 1H), 1.68-1.72 (m, 3H), 1.99-2.14 (m, 4H), 2.23-2.39 (m, 6H), 2.41-2.59 (m, 2H), 2.61-2.64 (m, 2H), 3.03-3.09 (m, 2H), 5.50-5.53 (br, 2H), 5.65-5.67 (br, 2H), 5.75 (s, 2H); ^{13}C NMR (100 MHz, $CDCl_3$) δ = 31.34, 35.79, 44.79, 46.77, 46.87, 48.77, 56.23, 61.14, 123.76, 127.09, 129.64, 225.99; IR υ_{max} = 1651, 1725, 2857, 2928 cm^{-1}; accurate mass (ESI, Q-ToF) *m/z*: calculated for $C_{23}H_{26}NaO_2$ $[M+Na]^+$ 357.1825, found: 357.1827.

Synthesis of compound 7

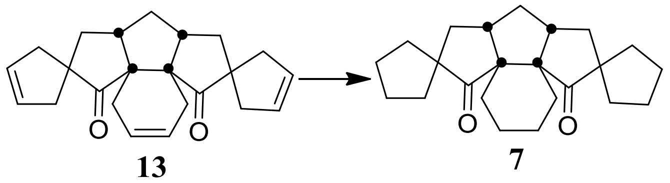

white solid, 90% yield (27.0 mg, starting with 30.0 mg of tris-RCM product **13**)

Mp 93-94 °C; R_f = 0.71 (silica gel, 10% EtOAc-petroleum ether): ^{1}H NMR (400 MHz, $CDCl_3$) δ = 1.25-1.30 (m, 2H), 1.32-1.80 (m, 21H), 1.81-1.94 (m, 4H), 2.11-2.18 (m, 2H),

2.36-2.53 (m, 3H); ^{13}C NMR (100 MHz, $CDCl_3$) δ = 21.72, 25.84, 26.03, 29.89, 36.03, 39.33, 41.64, 42.79, 45.04, 57.80, 62.25, 226.67; IR υ_{max} = 1727, 2862, 2938 cm^{-1}; accurate mass (ESI, Q-ToF) *m/z*: calculated for $C_{23}H_{32}KO_2$ $[M+K]^+$ 379.2034, found: 379.2038.

Synthesis of compound 14a

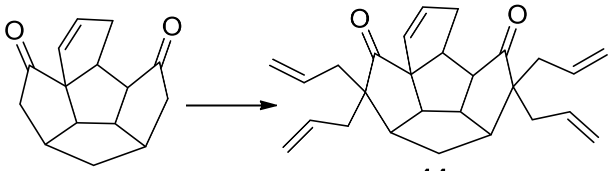

3a 14a

white solid, 74% (125.6 mg, starting with 100.0 mg of pentacyclic dione **3a**)

Mp 74-75 ^{o}C; R_f = 0.60 (silica gel, 10% EtOAc-petroleum ether): ^{1}H NMR (400 MHz, $CDCl_3$) δ = 0.73 (ABq, J = 12.8 Hz, 1H), 1.93-1.99 (m, 3H), 2.19-2.33 (m, 5H), 2.37-2.44 (m, 4H), 2.51 (t, J = 7.4 Hz, 1H), 2.66-2.69 (m, 1H), 2.85-2.90 (m, 1H), 3.19 (t, J = 8.0 Hz, 1H), 3.32-3.37 (m, 1H), 4.96-5.08 (m, 8H), 5.37-5.38 (m, 1H), 5.55-5.62 (m, 2H), 5.71-5.80 (m, 3H); ^{13}C NMR (100 MHz, $CDCl_3$) δ = 32.05, 35.21, 35.25, 38.97, 39.16, 42.51, 48.58, 49.94, 51.00, 54.14, 54.48, 58.39, 58.84, 62.73, 77.83, 118.31, 118.37, 118.40, 118.61, 132.25, 132.47, 132.78, 133.00, 133.82, 133.91, 220.60, 221.62; IR υ_{max} = 3075, 2927, 2854, 1730, 1639, 1440, 1270, 1150, cm^{-1}; accurate mass (ESI, Q-ToF) *m/z*: calculated for $C_{27}H_{33}O_2$ $[M+H]^+$ 389.2481, found: 389.2436.

Synthesis of compound 15a

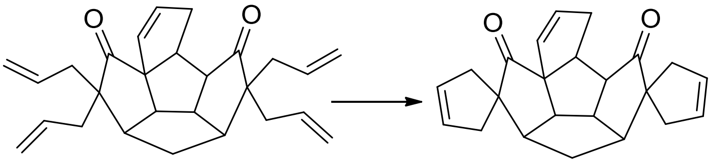

14a 15a

white solid, 92% (47.0 mg, starting with 60.0 mg of tetra-allyl pentacyclic dione **14a**)

Mp 203-205 ^{o}C; R_f = 0.38 (silica gel, 10% EtOAc-petroleum ether): ^{1}H NMR (400 MHz, $CDCl_3$) δ = 0.43 (ABq, J = 12.6 Hz, 1H), 1.61-1.65 (m, 1H), 1.90-1.96 (m, 1H), 2.18-2.24 (m, 2H), 2.31-2.48 (m, 5H), 2.52-2.58 (m, 3H), 2.70-2.76 (m, 2H), 2.79-2.82 (m, 1H), 3.18 (t, J = 8.0 Hz, 1H), 3.32-3.37 (m, 1H), 5.39-5.41 (m, 1H), 5.44-5.47 (m, 2H), 5.60-5.70 (m, 2H), 5.80-5.82 (m, 1H); ^{13}C NMR (125 MHz, $CDCl_3$) δ = 34.34 (t), 37.38 (t), 37.82 (t), 42.94 (t), 45.14 (t), 45.34 (t), 49.48 (d), 52.67 (d), 53.58 (d), 54.36 (d), 55.16 (d), 61.48 (d), 61.65 (s), 61.86 (s), 126.08 (d), 126.28 (d), 130.50 (d), 130.54 (d), 132.24 (d), 133.02 (d), 220.97 (s), 221.66 (s); IR υ_{max} = 3054, 2922, 2837, 1723, 1627, 1435, 1271, 1151,1107, 1017 cm^{-1};

accurate mass (ESI, Q-ToF) *m/z*: calculated for $C_{23}H_{24}NaO_2$ $[M+Na]^+$ 355.1669, found: 355.1669.

Synthesis of compound 16a

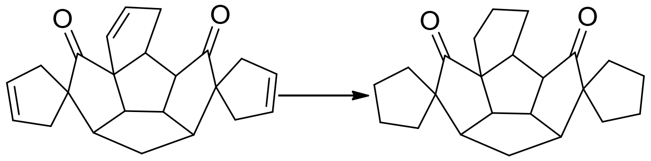

15a 16a

white solid, 98% (29.90 mg, starting with 30.0 mg of bis-RCM product **15a**)

Mp 163-165 °C; R_f = 0.63 (silica gel, 10% EtOAc-petroleum ether): ^{1}H NMR (500 MHz, $CDCl_3$) δ = 0.36 (ABq, *J* = 12.7 Hz, 1H), 1.34-1.46 (m, 3H), 1.51-1.62 (m, 4H), 1.64-1.95 (m, 15H), 2.32-2.45 (m, 4H), 2.61-2.65 (m, 1H), 2.97 (t, *J* = 8.6 Hz, 1H), 3.31-3.38 (m, 1H); ^{13}C NMR (125 MHz, $CDCl_3$) δ = 25.62 (t), 25.69 (t), 25.94 (t), 26.77 (t), 30.35 (t), 30.82 (t), 34.45 (t), 37.61 (t), 38.43 (t), 38.77 (t), 42.71 (t), 50.45 (d), 52.64 (d), 53.26 (d), 56.64 (d), 59.06 (d), 60.72 (d), 63.31 (s), 63.51 (s), 69.60 (s), 223.05 (s), 226.43 (s); IR υ_{max} = 2928, 2861, 1725, 1627, 1459, 1268, 1122, 1073, 1044 cm^{-1}; accurate mass (ESI, Q-ToF) *m/z*: calculated for $C_{23}H_{30}NaO_2$ $[M+Na]^+$ 361.2138, found: 361.2131.

Synthesis of compound 14b

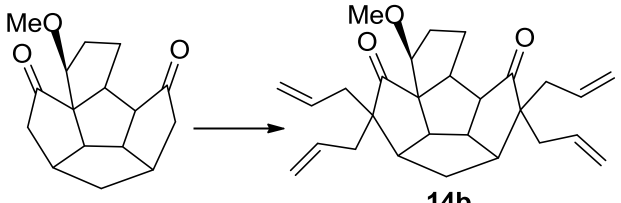

3b 14b

white solid, 60% (65.0 mg, starting with 67.0 mg of pentacyclic dione **3b**)

Mp 149-151 °C; R_f = 0.40 (silica gel, 10% EtOAc-petroleum ether): ^{1}H NMR (400 MHz, $CDCl_3$) δ = 0.55 (ABq, *J* = 12.8 Hz, 1H), 1.42-1.55 (m, 1H), 1.58-1.64 (m, 1H), 1.83-1.94 (m, 4H), 2.04-2.17 (m, 2H), 2.20-2.22 (d, *J* = 7.4 Hz, 2H), 2.29-2.46 (m, 6H), 2.57-2.60 (m, 1H), 3.31 (s, 3H), 3.38-3.44 (m, 1H), 3.51-3.54 (m, 1H), 3.60 (t, *J* = 8.3 Hz, 1H), 4.92-5.09 (m, 8H), 5.51-5.62 (m, 2H), 5.70-5.80 (m, 2H); ^{13}C NMR (100 MHz, $CDCl_3$) δ = 30.73 (t), 31.98 (t), 32.24 (t), 34.53 (t), 34.95 (t), 36.88 (t), 38.84 (t), 48.84 (d), 50.06 (d), 50.26 (d), 51.09 (d), 56.30 (d), 57.74 (q), 58.74 (s), 59.13 (s), 61.66 (d), 73.01 (s), 86.97 (d), 118.15 (t), 118.27 (t), 118.31 (t), 133.09 (d), 133.28 (d), 134.01 (d), 221.34 (s), 223.00 (s); IR υ_{max} = 3073, 2923, 1724, 1638, 1441, 1266, 1161, 1109, 1020 cm^{-1}; accurate mass (ESI, Q-ToF) *m/z*: calculated for $C_{28}H_{37}O_3$ $[M+H]^+$ 421.2743, found: 421.2761.

Synthesis of compound 15b

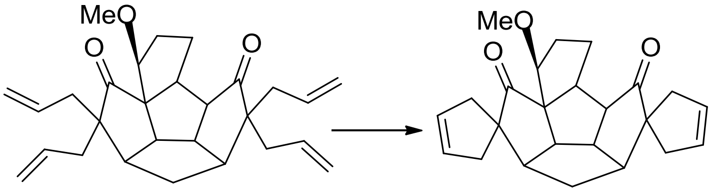

14b 15b

white solid, 92% (40.0 mg, starting with 50.0 mg of tetra-allyl pentacyclic dione **14b**)

Mp 138-140 °C; R_f = 0.38 (silica gel, 10% EtOAc-petroleum ether): ^{1}H NMR (500 MHz, $CDCl_3$) δ = 0.27 (ABq, J = 12.8 Hz, 1H), 1.54-1.64 (m, 2H), 1.86-1.95 (m, 2H), 2.06-2.11 (m, 1H), 2.14-2.20 (m, 2H), 2.20-2.24 (m, 5H), 2.48-2.55 (m, 2H), 2.67-2.72 (m, 3H), 3.32 (s, 3H), 3.36-3.42 (m, 1H), 3.58-3.63 (m, 2H), 5.43-5.45 (m, 2H), 5.66-5.67 (m, 2H); ^{13}C NMR (125 MHz, $CDCl_3$) δ = 30.93 (t), 32.13 (t), 34.65 (t), 37.02 (t), 37.90 (t), 44.55 (t), 45.16 (t), 49.80 (d), 51.04 (d), 52.77 (d), 53.42 (d), 56.04 (d), 57.91 (q), 60.69 (d), 61.77 (s), 61.87 (s), 72.60 (s), 88.19 (d), 126.29 (d), 126.48 (d), 130.27 (d), 130.50 (d), 221.07 (s), 224.30 (s); IR υ_{max} = 3057, 2927, 2854, 1731, 1422, 1265, 1114, 1044 cm^{-1}; accurate mass (ESI, Q-ToF) *m/z*: calculated for $C_{24}H_{29}O_3$ $[M+H]^+$ 365.2100, found: 365.2064.

Synthesis of compound 16b

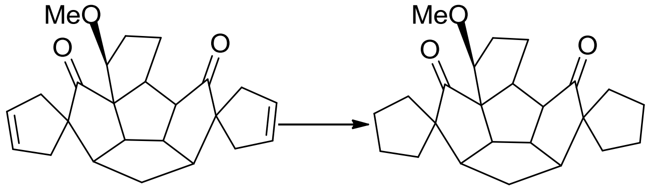

15b 16b

white solid, 99% (30.0 mg, starting with 30.0 mg of bis-RCM product **15b**)

Mp 112-114 °C; R_f = 0.48 (silica gel, 10% EtOAc-petroleum ether): ^{1}H NMR (400 MHz, $CDCl_3$) δ = 0.36 (ABq, J = 12.7 Hz, 1H), 1.33-1.45 (m, 2H), 1.49-1.64 (m, 12H), 1.67-1.77 (m, 2H), 1.80-1.91 (m, 4H), 2.04-2.10 (m, 1H), 2.33-2.42 (m, 3H), 2.60-2.65 (m, 1H), 3.29 (s, 3H), 3.32-3.37 (m, 1H), 3.47-3.60 (m, 2H); ^{13}C NMR (100 MHz, $CDCl_3$) δ = 25.51 (t), 25.68 (t), 25.86 (t), 25.94 (t), 30.33 (t), 30.99 (t), 31.26 (t), 31.89 (t), 34.79 (t), 38.28 (t), 38.66 (t), 50.06 (d), 51.26 (d), 52.16 (d), 53.29 (d), 56.08 (d), 57.95 (q), 61.07 (d), 63.53 (s), 63.74 (s), 72.77 (s), 87.98 (d), 223.04 (s), 225.98 (s); IR υ_{max} = 2927, 2867, 1724, 1449, 1266, 1115, 1087 cm^{-1}; accurate mass (ESI, Q-ToF) *m/z*: calculated for $C_{24}H_{33}O_3$ $[M+H]^+$ 369.2400, found: 369.2373.

Synthesis of compound 14c

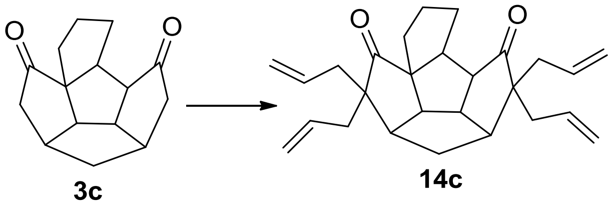

Colorless semi-solid, 70% (95.0 mg, starting with 80.0 mg of pentacyclic dione **3c**)

R_f = 0.56 (silica gel, 10% EtOAc-petroleum ether): ^{1}H NMR (400 MHz, $CDCl_3$) δ = 0.58 (ABq, J = 12.8 Hz, 1H), 1.53-1.62 (m, 1H), 1.65-1.72 (m, 2H), 1.73-1.84 (m, 2H), 1.84-1.97 (m, 5H), 2.23 (t, J = 7.0 Hz, 4H), 2.32-2.43 (m, 3H), 2.44-2.50 (m, 1H), 2.58-2.62 (m, 1H), 3.04 (t, J = 8.2 Hz, 1H), 3.40-3.47 (m, 1H), 4.95-5.19 (m, 8H), 5.52-5.65 (m, 2H), 5.70-5.80 (m, 2H); ^{13}C NMR (100 MHz, $CDCl_3$) δ = 26.83, 31.90, 34.80, 34.95, 37.49, 38.86, 38.95, 42.33, 49.46, 50.50, 51.00, 57.11, 57.81, 58.64, 58.98, 61.16, 69.80, 118.30, 118.33, 118.45, 133.02, 133.09, 134.01, 221.28, 224.09; IR υ_{max} = 2955, 2928, 2857, 1731, 1464, 1380, 1274, 1124, 1073 cm^{-1}; accurate mass (ESI, Q-ToF) *m/z*: calculated for $C_{27}H_{35}O_2$ $[M+H]^+$ 391.2637, found: 391.2795.

Synthesis of compound 15c

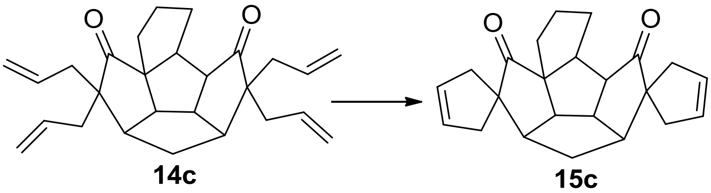

white solid, 91% (32.0 mg, starting with 41.0 mg of tetra-allyl pentacyclic dione **14c**)

Mp 175-177 ^oC; R_f = 0.50 (silica gel, 10% EtOAc-petrolium ether): ^{1}H NMR (500 MHz, $CDCl_3$) δ = 0.26 (ABq, J = 12.8 Hz, 1H), 1.57-1.63 (m, 1H), 1.64-1.71 (m, 2H), 1.76-1.83 (m, 2H), 1.84-1.97 (m, 3H), 2.12-2.20 (m, 2H), 2.32-2.45 (m, 3H), 2.48-2.61 (m, 3H), 2.68-2.75 (m, 3H), 3.04 (q, J = 8.2 Hz, 1H), 3.37-3.43 (m, 1H), 5.43-5.46 (m, 2H), 5.66-5.70 (m, 2H); ^{13}C NMR (125 MHz, $CDCl_3$) δ = 27.00 (t), 34.25 (t), 37.02 (t), 37.50 (t), 37.89 (t), 43.26 (t), 44.69 (t), 45.20 (t), 50.20 (d), 53.01 (d), 53.36 (d), 56.53 (d), 58.82 (d), 60.34 (d), 61.56 (s), 61.64 (s), 69.41 (s), 126.17 (d), 126.26 (d), 130.55 (d), 130.56 (d), 221.00 (s), 224.58 (s); IR υ_{max} = 3054, 2925, 2854, 1724, 1622, 1459, 1269, 1124 cm^{-1}; accurate mass (ESI, Q-ToF) *m/z*: calculated for $C_{23}H_{27}O_2$ $[M+H]^+$ 335.2011, found: 335.1969.

Synthesis of compound 14d

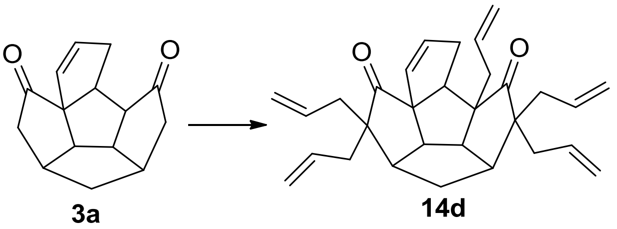

white solid, 67% (127 mg, starting with 100 mg of pentacyclic dione **3a**)

Mp 101-103 °C; R_f = 0.69 (silica gel, 10% EtOAc-petroleum ether): ^{1}H NMR (500 MHz, $CDCl_3$) δ = 0.99, 1.02 (ABq, J = 14.6 Hz, 1H), 1.99-2.61 (m, 16H), 3.21-3.28 (m, 2H), 4.98-5.47 (m, 10H), 5.46-5.47 (br, 1H), 5.53-5.62 (m, 3H), 5.76-5.81 (m, 2H), 5.89-5.91 (br, 1H); ^{13}C NMR (100.8 MHz, $CDCl_3$) δ = 32.63, 34.28, 34.70, 35.84, 36.84, 37.86, 38.65, 50.26, 50.79, 52.84, 53.00, 56.83, 58.07, 59.30, 65.10, 118.43, 118.52, 118.57, 118.64, 118.81, 132.75, 132.91, 133.39, 133.84, 134.01, 134.66, 135.19, 220.24, 222.72; IR υ_{max} = 1639, 1727, 2930 cm^{-1}; accurate mass (ESI, Q-ToF) *m/z*: calculated for $C_{30}H_{36}NaO_2$ [M+Na]$^+$ 451.2608 found: 451.2610.

Synthesis of compound 15d

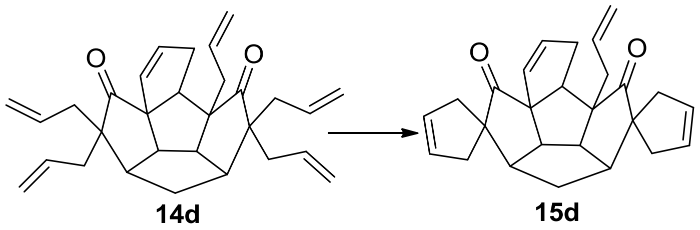

white solid, 85% (44 mg, starting with 60 mg of peta-allyl pentacyclic dione **14d**)

Mp 116-118 °C; R_f = 0.66 (silica gel, 10% EtOAc-petroleum ether): ^{1}H NMR (500 MHz, $CDCl_3$) δ = 0.64, 0.70 (ABq, J = 12.7 Hz, 1H), 1.62-1.99 (m, 1H), 2.16-2.30 (m, 4H), 2.37-2.50 (m, 3H), 2.51-2.69 (m, 6H), 2.72-2.80 (m, 2H), 3.20-3.29 (m, 2H), 5.01-5.06 (m, 2H), 5.45-5.49 (m, 3H), 5.53-5.59 (m, 1H), 5.61-5.72 (m, 2H), 5.89-5.92 (m, 1H); ^{13}C NMR (100.8 MHz, $CDCl_3$) δ = 34.39, 35.40, 36.98, 37.32, 39.44, 44.17, 47.37, 52.46, 52.86, 53.79, 53.84, 57.74, 61.31, 61.97, 65.15, 76.34, 118.62, 126.06, 126.48, 129.77, 130.58, 134.13, 135.20, 220.11, 225.18; IR υ_{max} = 1621, 1637, 1730, 2845, 2922 cm^{-1}; accurate mass (ESI, Q-ToF) *m/z*: calculated for $C_{26}H_{28}NaO_2$ [M+Na]$^+$ 395.1982, found: 395.1983.

Synthesis of compound 16d

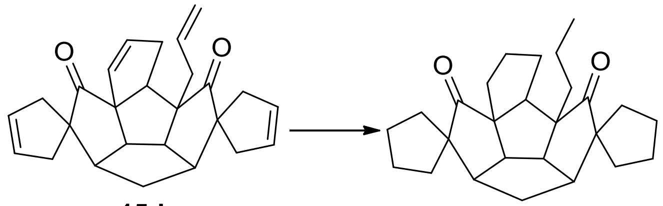

white solid, 97% (33 mg, starting with 35 mg of bis-RCM product **16d**)

Mp 135-136 °C; R_f = 0.68 (silica gel, 10% EtOAc-petroleum ether): ^{1}H NMR (500 MHz, $CDCl_3$) δ = 0.71, 0.77 (ABq, J = 12.6 Hz, 1H), 0.88-0.92 (m, 3H), 1.02-1.07 (m, 1H), 1.20-1.27 (m, 2H), 1.77-1.95 (m, 24H), 2.24 (d, J = 8.0 Hz, 1H), 2.37-2.44 (m, 2H), 3.01 (t, J = 7.8 Hz, 1H), 3.21 (t, J = 8.4 Hz, 1H); ^{13}C NMR (100.8 MHz, $CDCl_3$) δ = 15.04, 19.65, 25.42, 25.57, 25.72, 25.89, 27.19, 27.54, 30.87, 32.98, 35.11, 35.64, 38.83, 39.52, 40.12, 51.56, 52.31, 55.92, 55.97, 59.41, 62.59, 64.32, 65.24, 69.91, 226.13; IR υ_{max} = 1720, 2869, 2956 cm^{-1}; accurate mass (ESI, Q-ToF) *m/z*: calculated for $C_{26}H_{36}NaO_2$ $[M+Na]^+$ 403.2608, found: 403.2608.

References

[1] Gore, S.; Baskaran, S.; König, B. *Org. Lett.* **2012**, *14*, 4568. doi**:** 10.1021/ol302034r.

[2] Kotha, S.; Chinnam, A. K. *Synthesis* **2014**, 301. doi: 10.1055/s-0033-1340341.

[3] Kotha, S.; Chinnam, A. K.; Tiwari, A. *Beilstein J. Org. Chem.* **2013**, *9*, 2709. doi:10.3762/bjoc.9.307.

[4] Kotha, S.; Ali, R.; Chinnam, A. K. *Tetrahedron Lett.* **2014**, *55*, 4492. doi:10.1016/j.tetlet.2014.06.049.
