# Supplementary material for: Hybrid macrocycle formation and spiro annulation on cis-syn-cis-tricyclo[6.3.0.02,6]undeca-3,11-dione and its congeners via ring-closing metathesis
Source: Beilstein J Org Chem. 2015 Jul 6;11:1123–8. doi: 10.3762/bjoc.11.126 (PMC4505178; doi:10.3762/bjoc.11.126)

Supporting Information File 2

for

Hybrid macrocycle formation and spiro annulation on *cis-syn-cis*-tricyclo[6.3.0.0^{2,6}]undeca-3,11-dione and its congeners via ring-closing metathesis

Sambasivarao Kotha*, Ajay Kumar Chinnam and Rashid Ali

Address: Department of Chemistry, Indian Institute of Technology-Bombay, Powai, India, Fax: 022-2572 7152

Email: Sambasivarao Kotha - srk@chem.iitb.ac.in

*Corresponding author

NMR spectra

Compound 8 ^{1}H and ^{13}C NMR (400 MHz, $CDCl_3$)

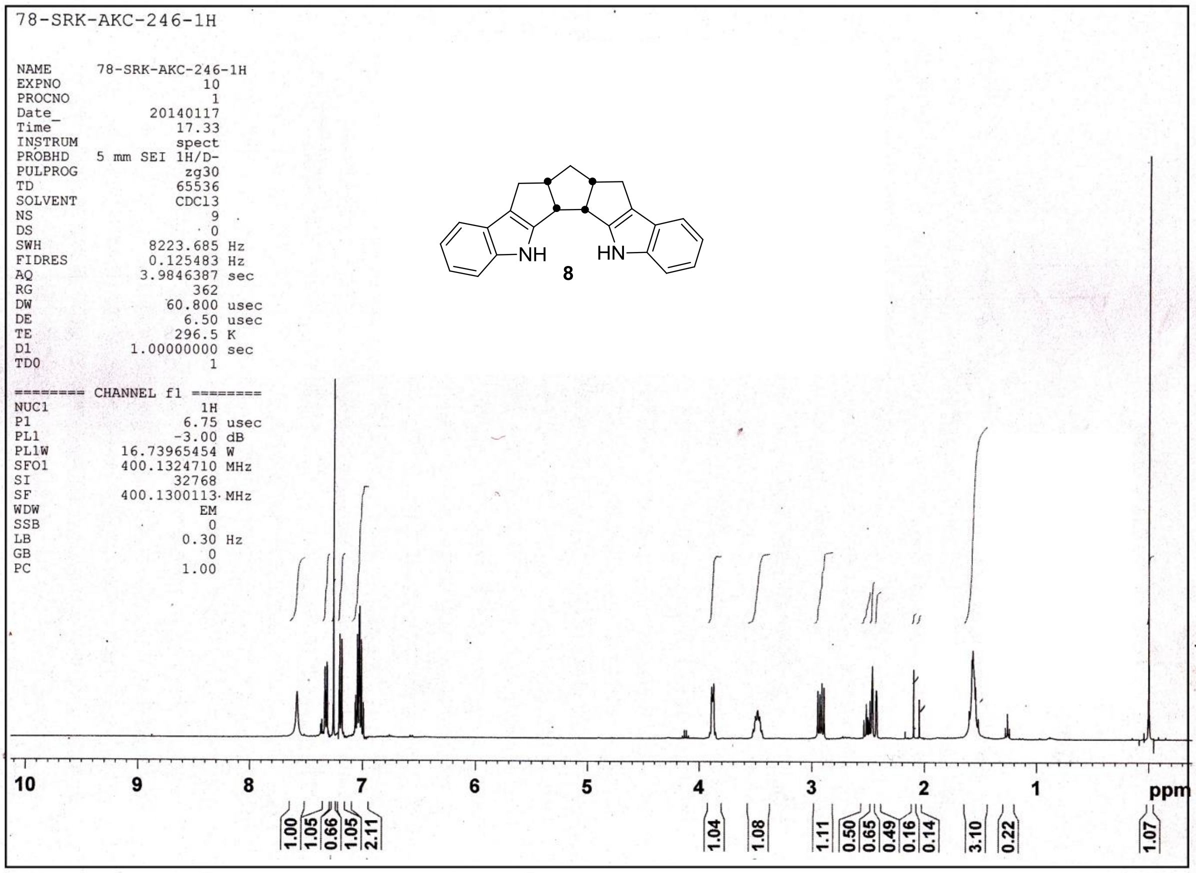

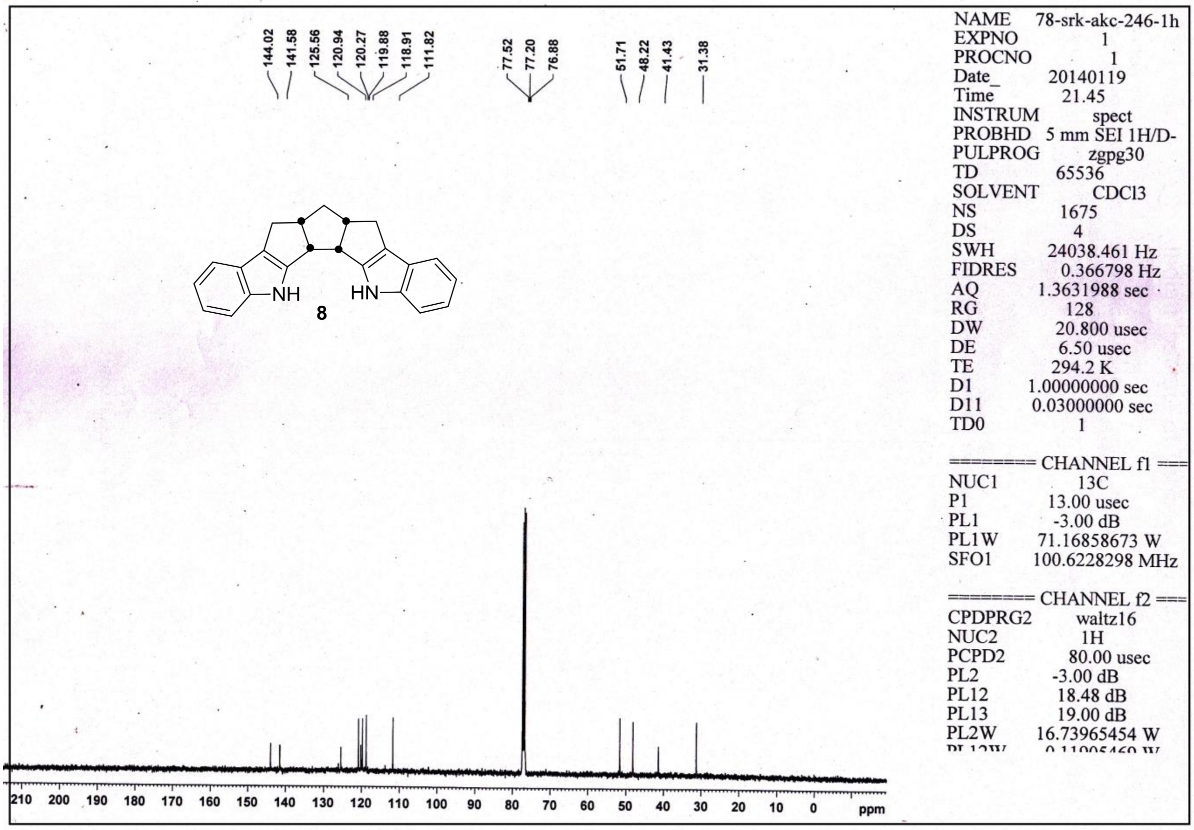

Compound 9 ^{1}H and ^{13}C NMR (400 MHz, $CDCl_3$)

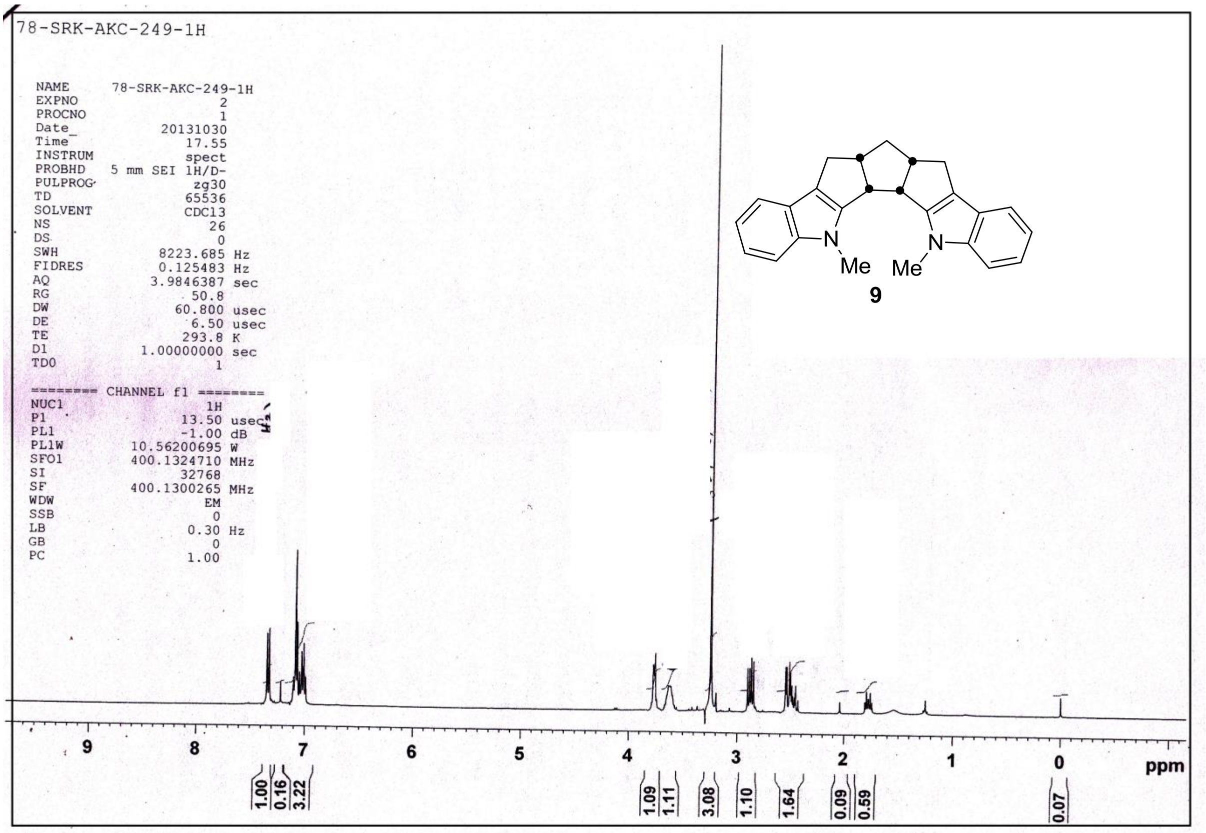

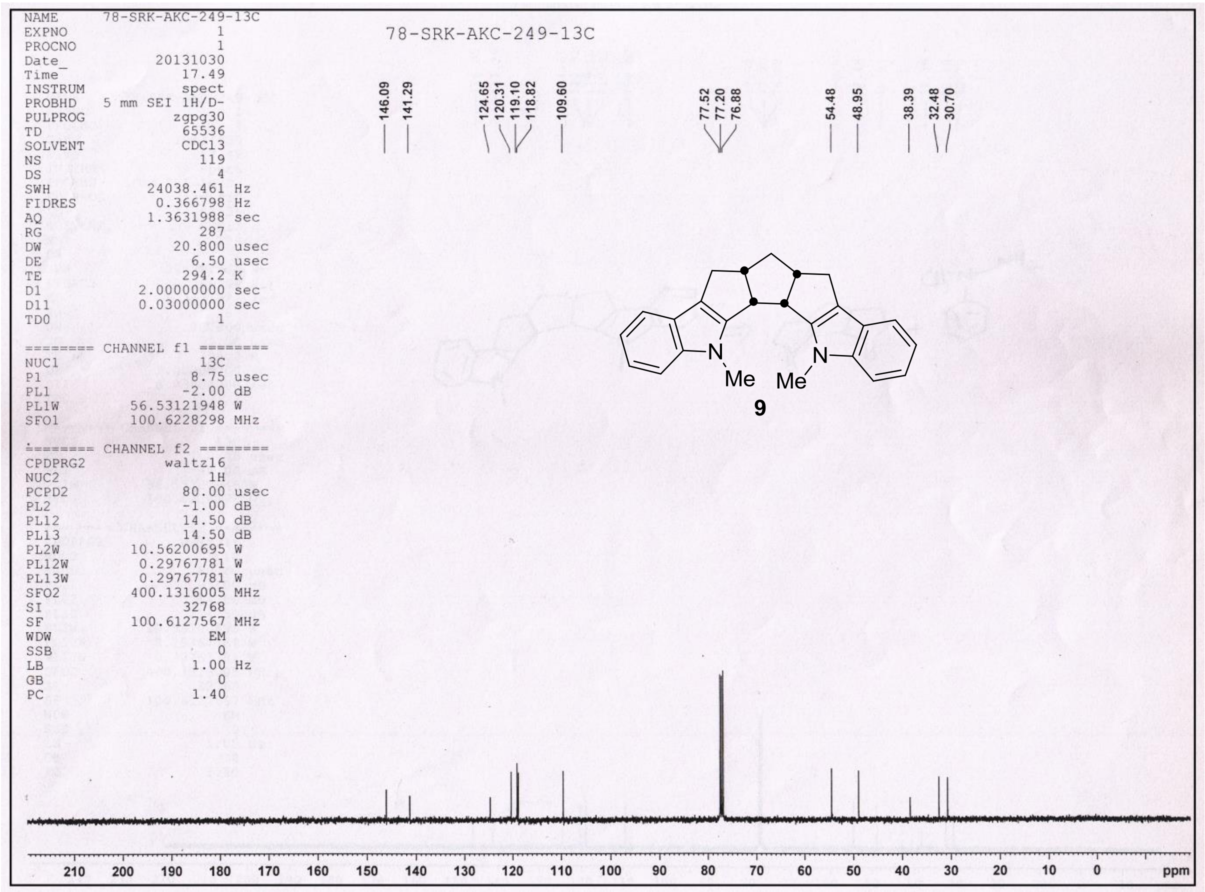

Compound 10 ^{1}H and ^{13}C NMR (500 MHz, $CDCl_3$)

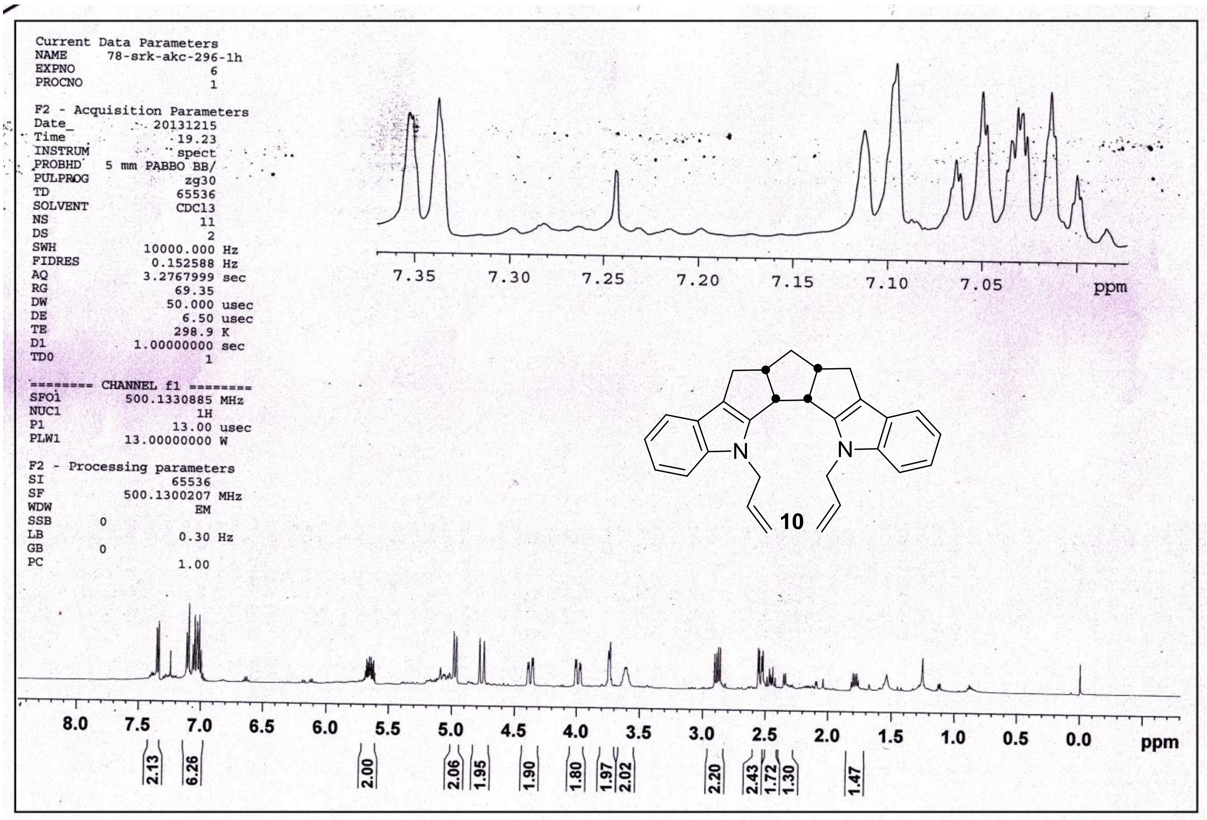

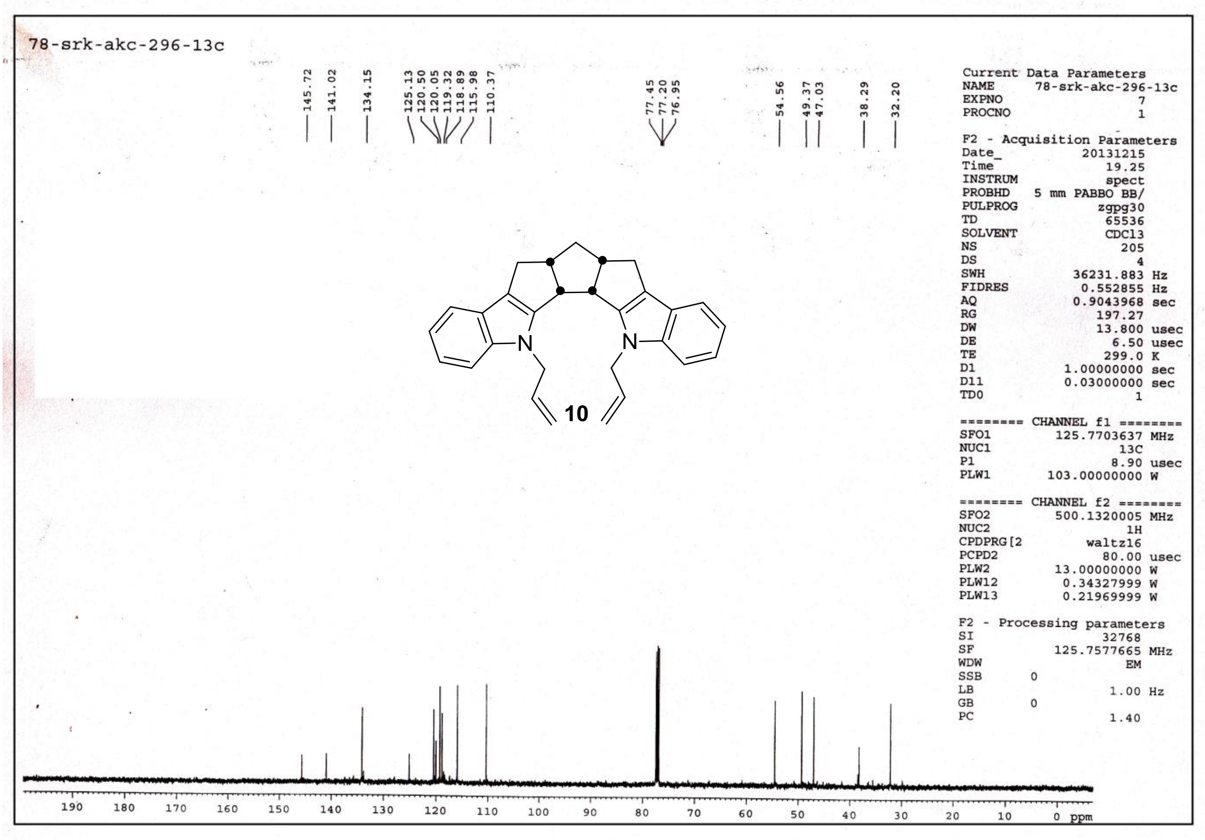

Compound 11 ^{1}H and ^{13}C NMR (400 MHz, $CDCl_3$)

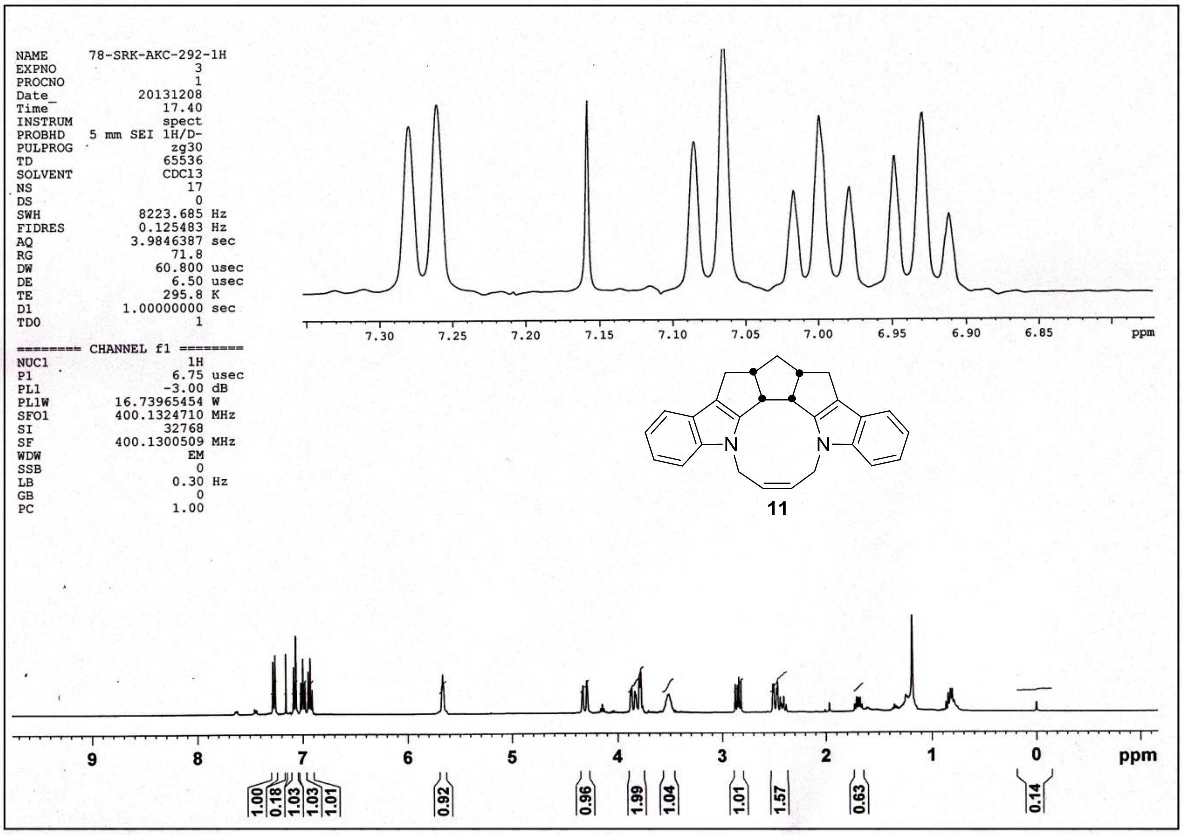

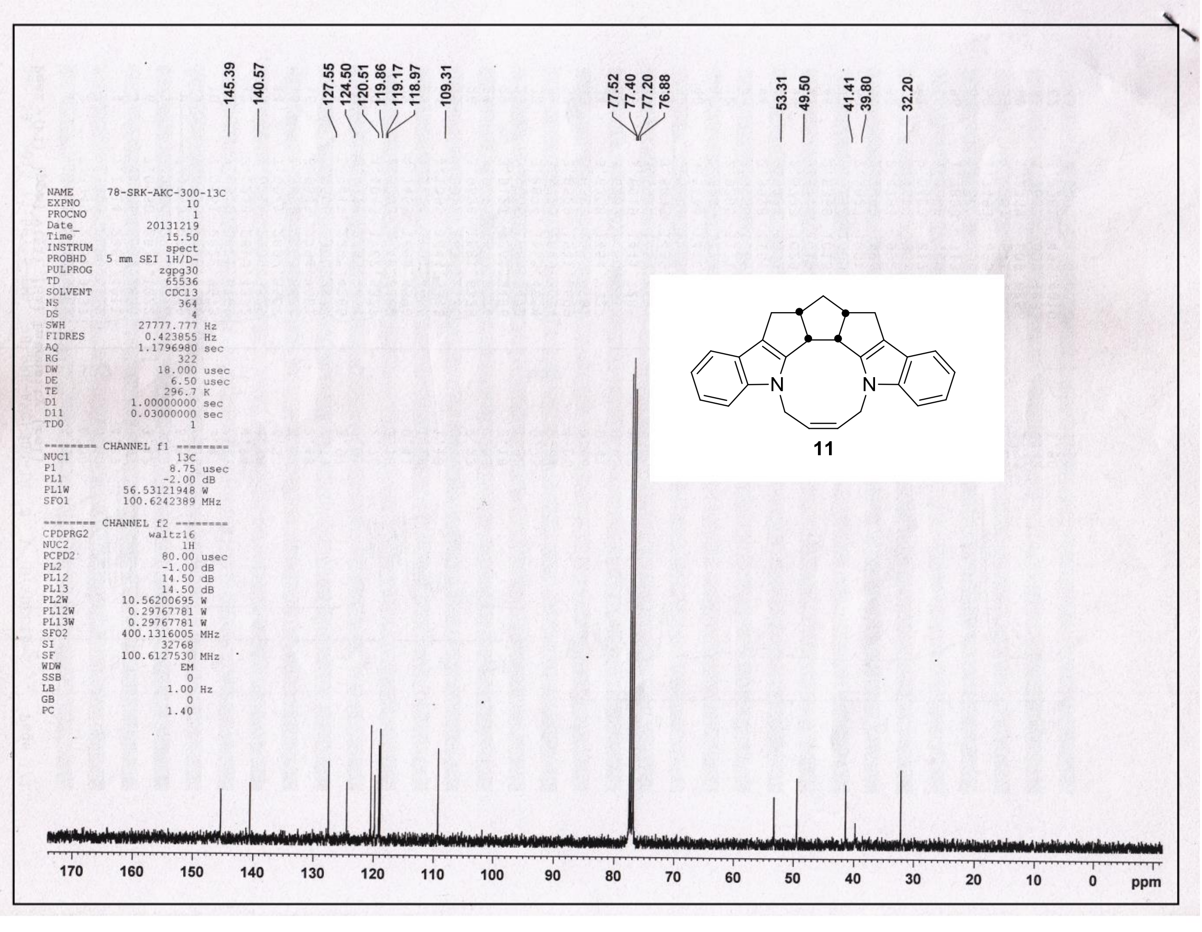

Compound 6 ^{1}H (400 MHz, $CDCl_3$) and ^{13}C NMR (500 MHz, $CDCl_3$)

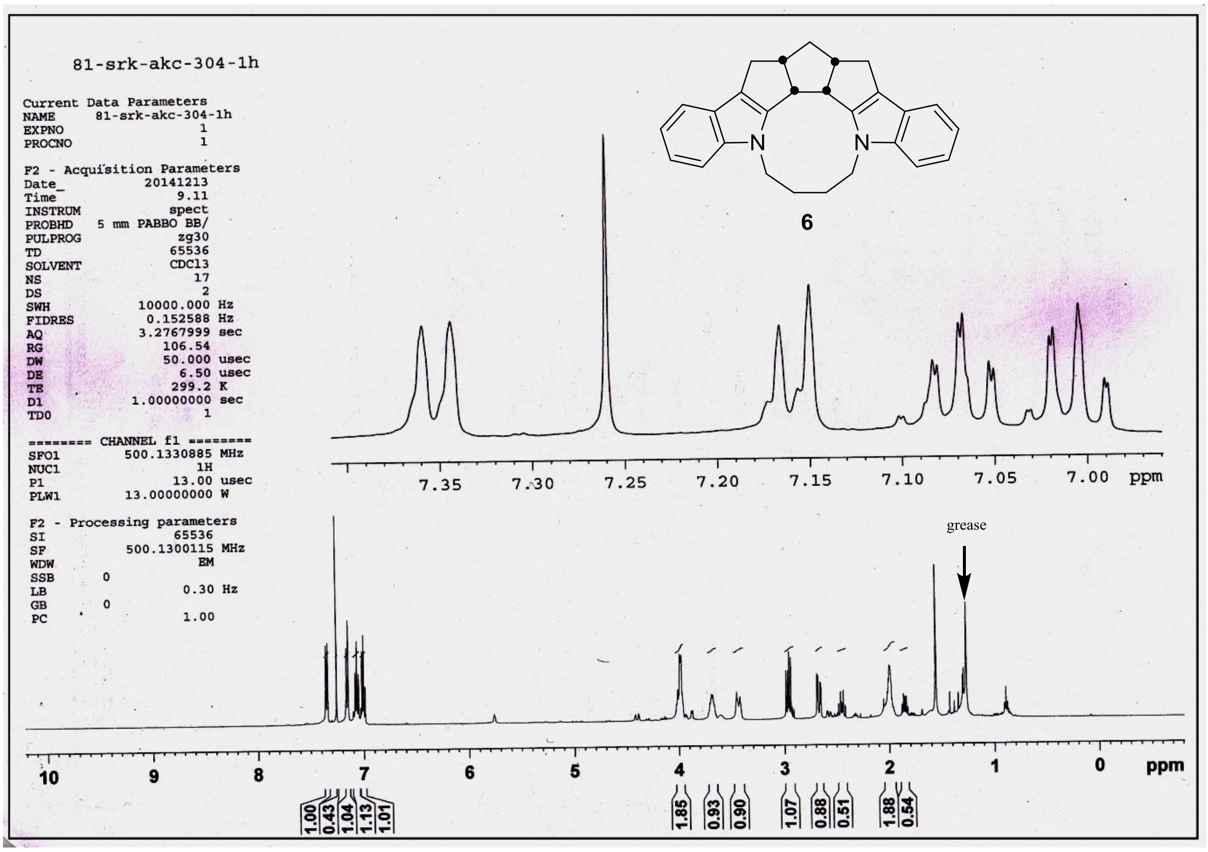

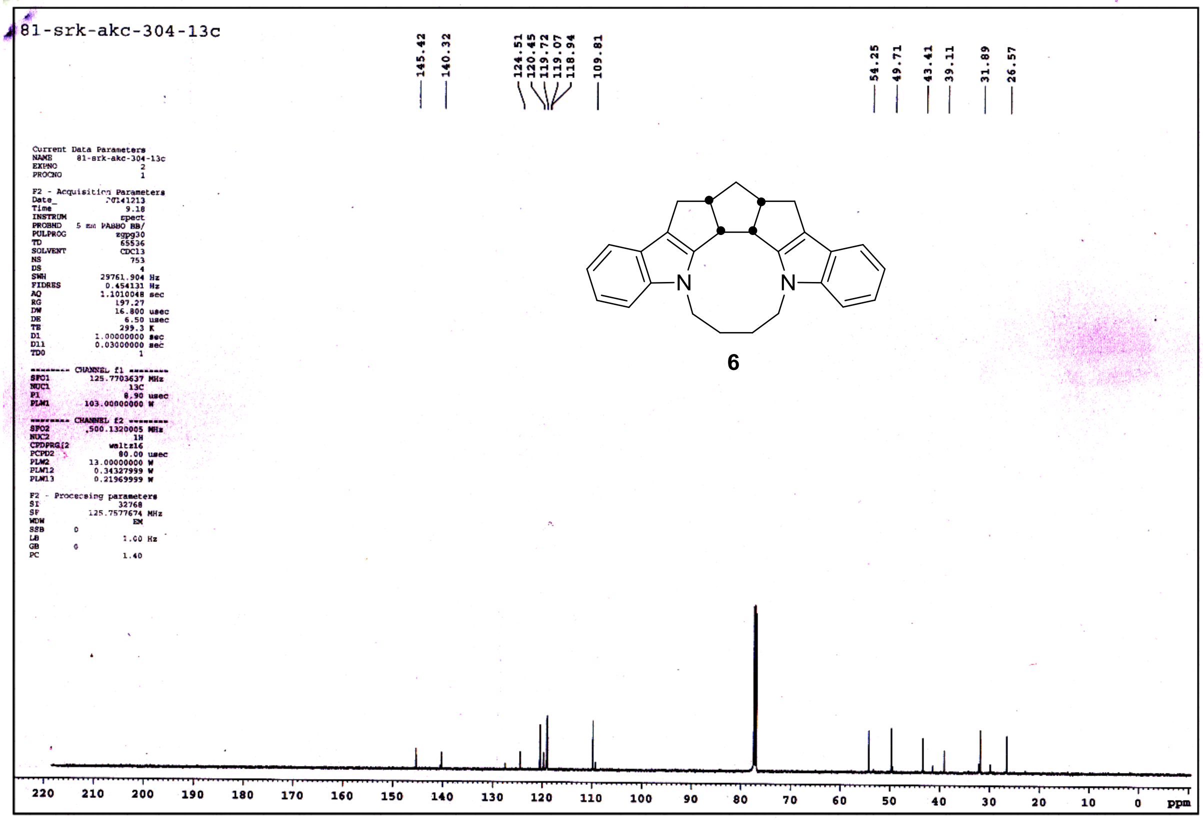

Compound 12 ^{1}H and ^{13}C NMR (400 MHz, $CDCl_3$)

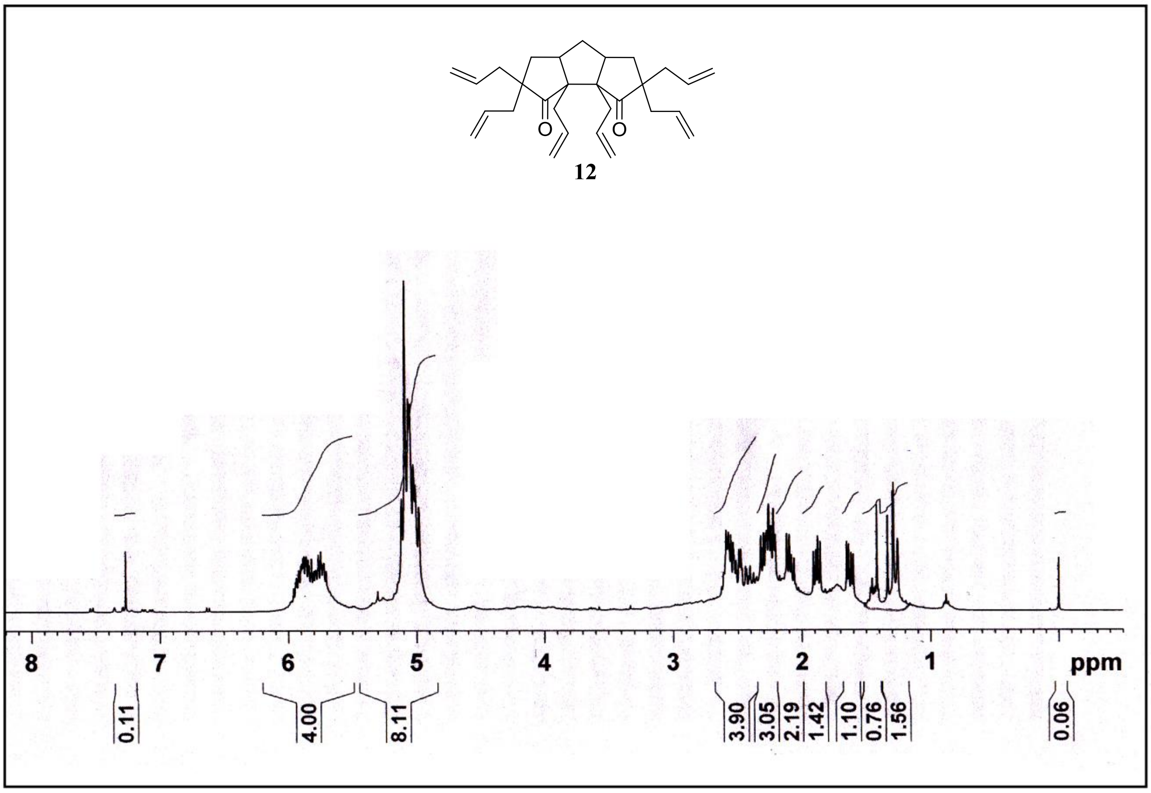

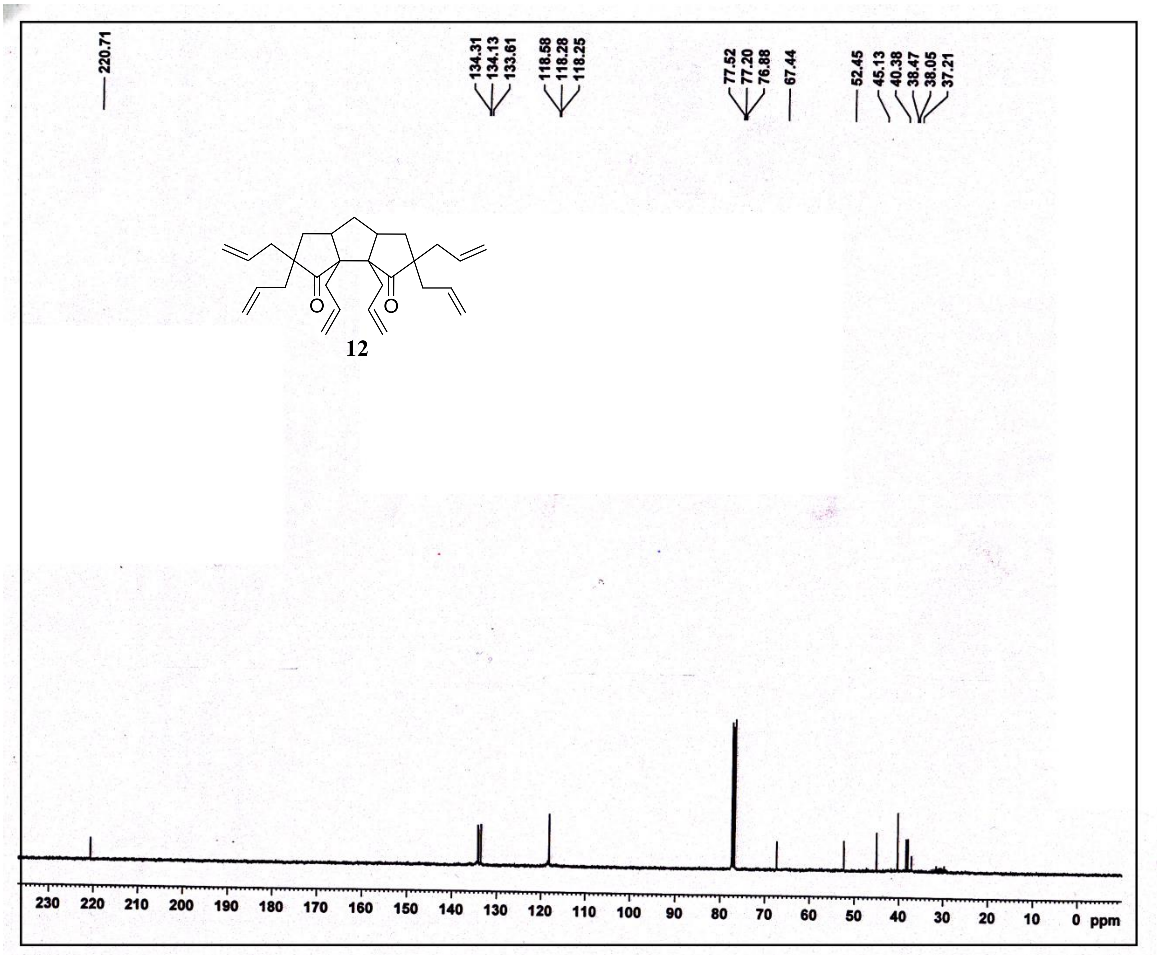

Compound 13 ^{1}H and ^{13}C NMR (400 MHz, $CDCl_3$)

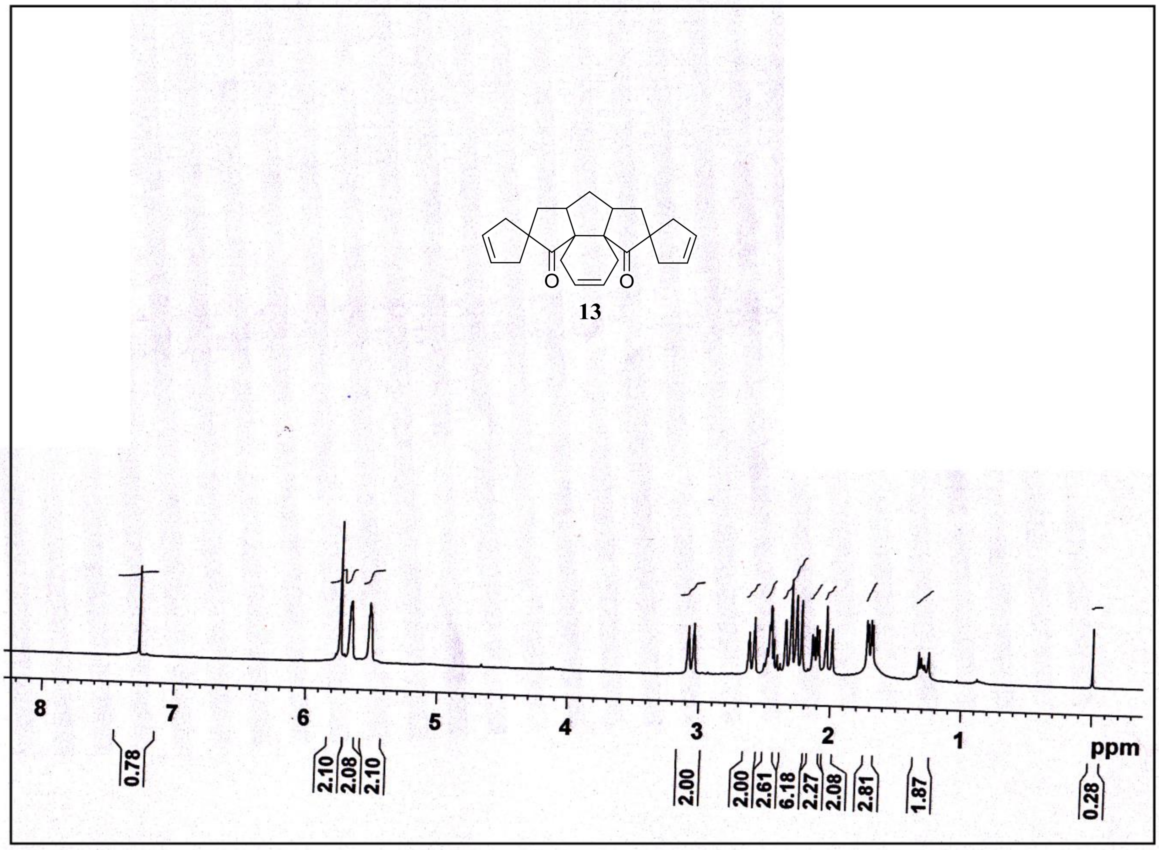

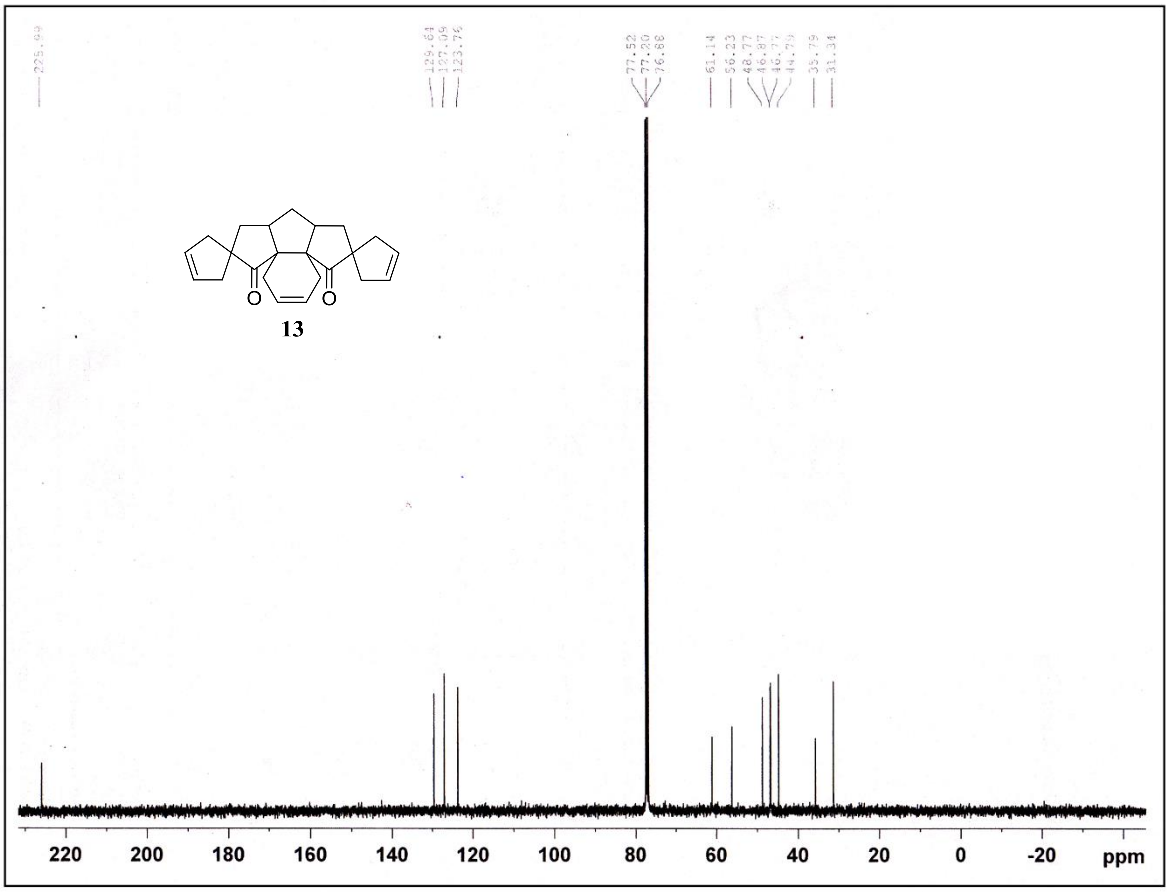

Compound 7 ^{1}H and ^{13}C NMR (400 MHz, $CDCl_3$)

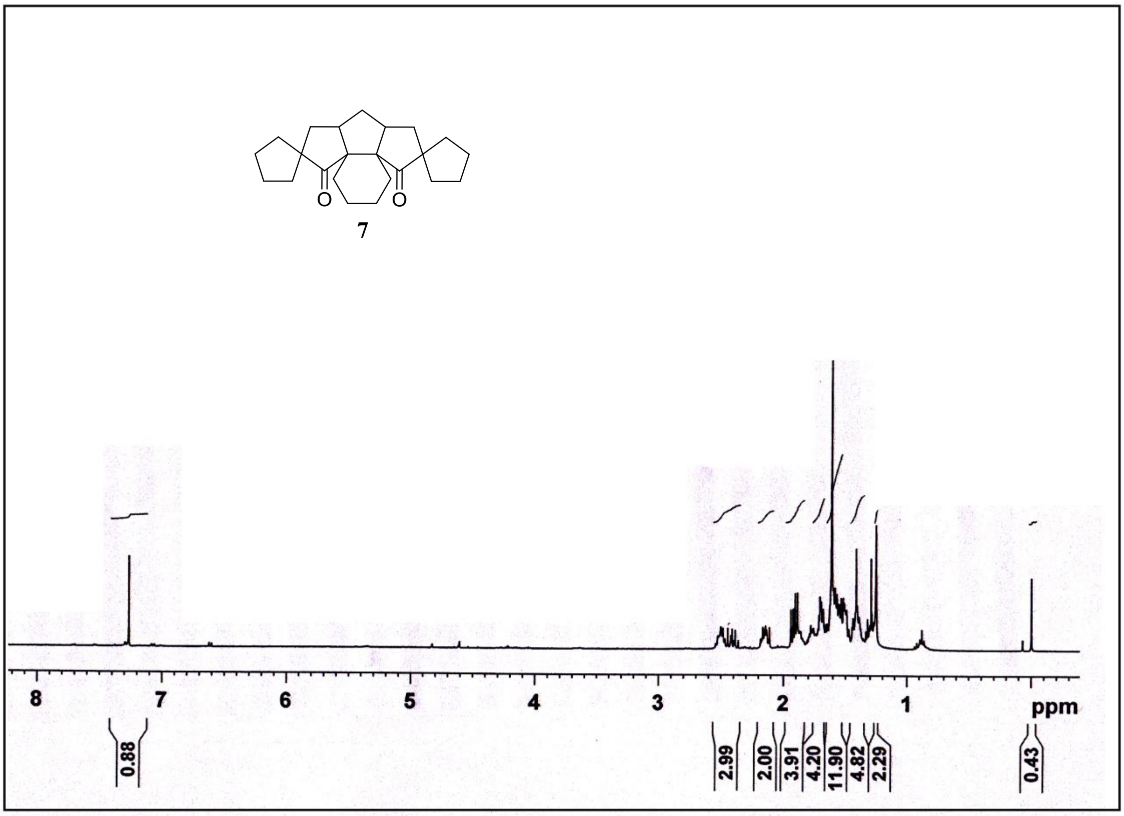

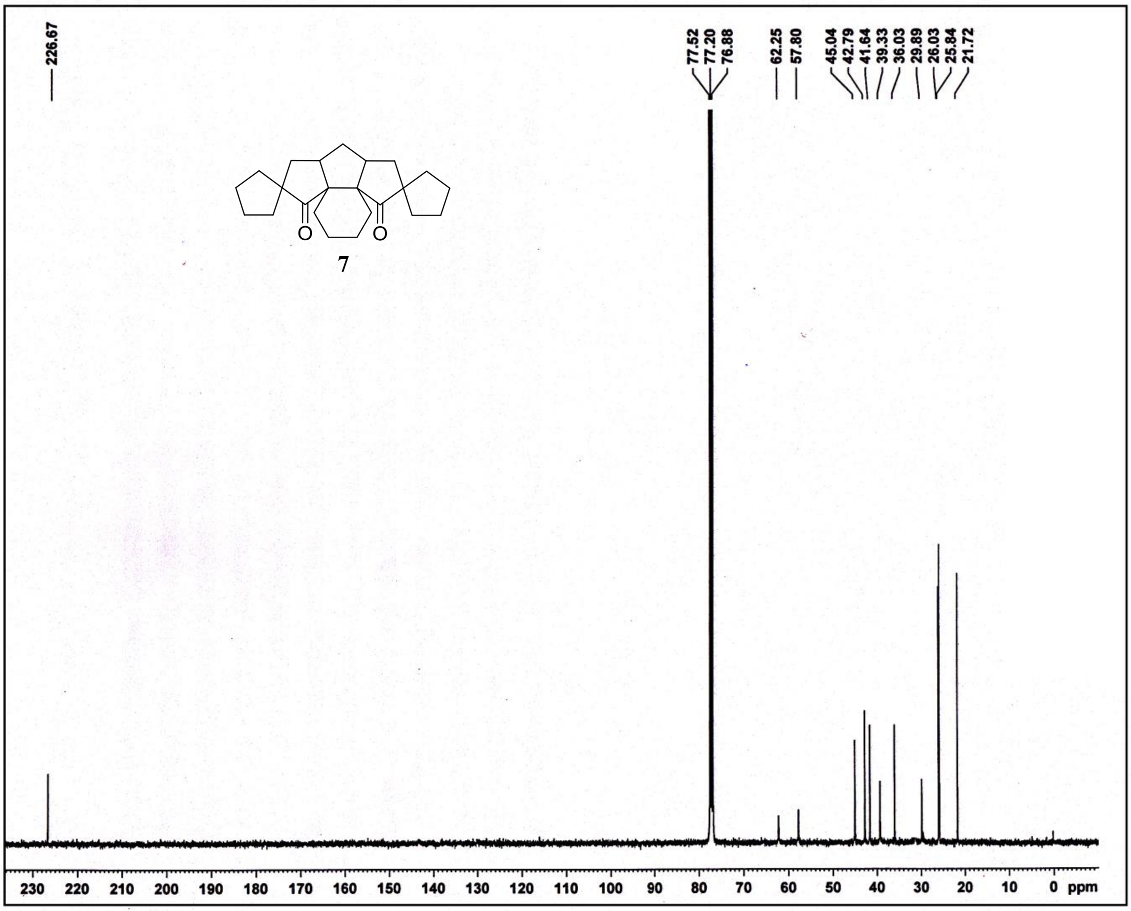

Compound 14a ^{1}H and ^{13}C NMR (400 MHz, CDCl$_3$)

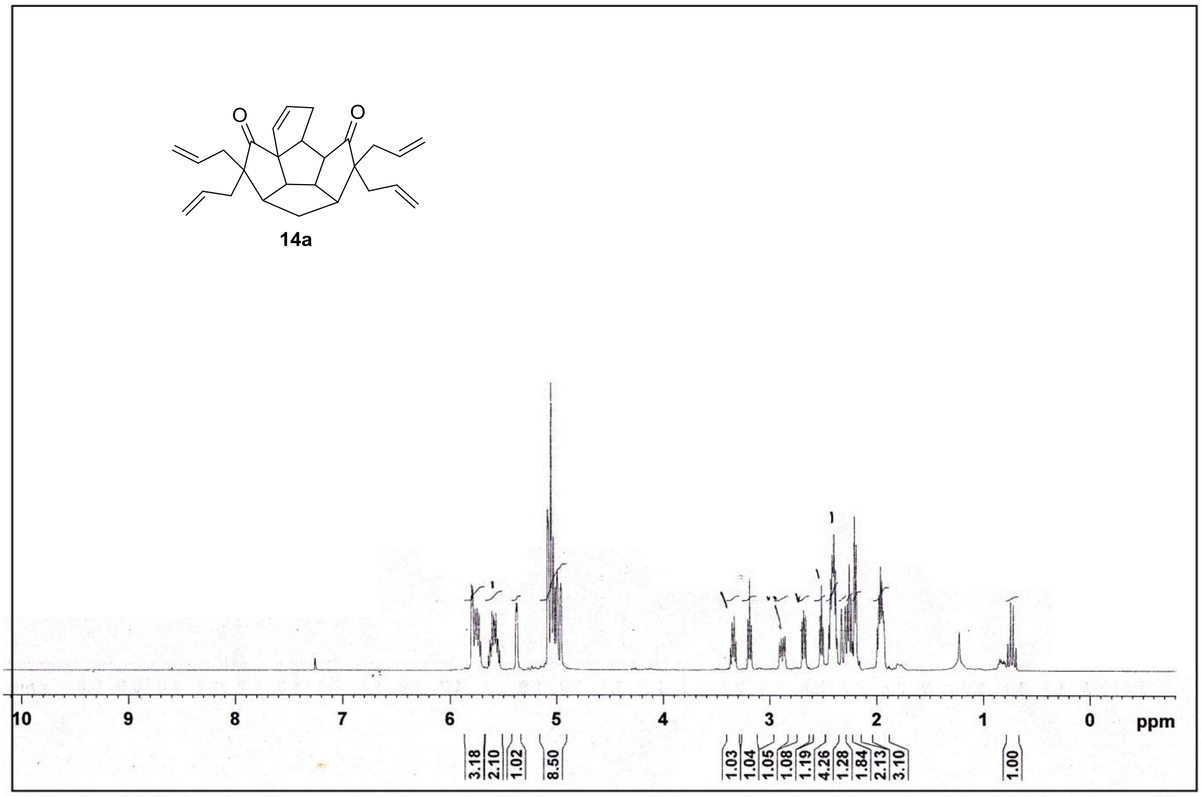

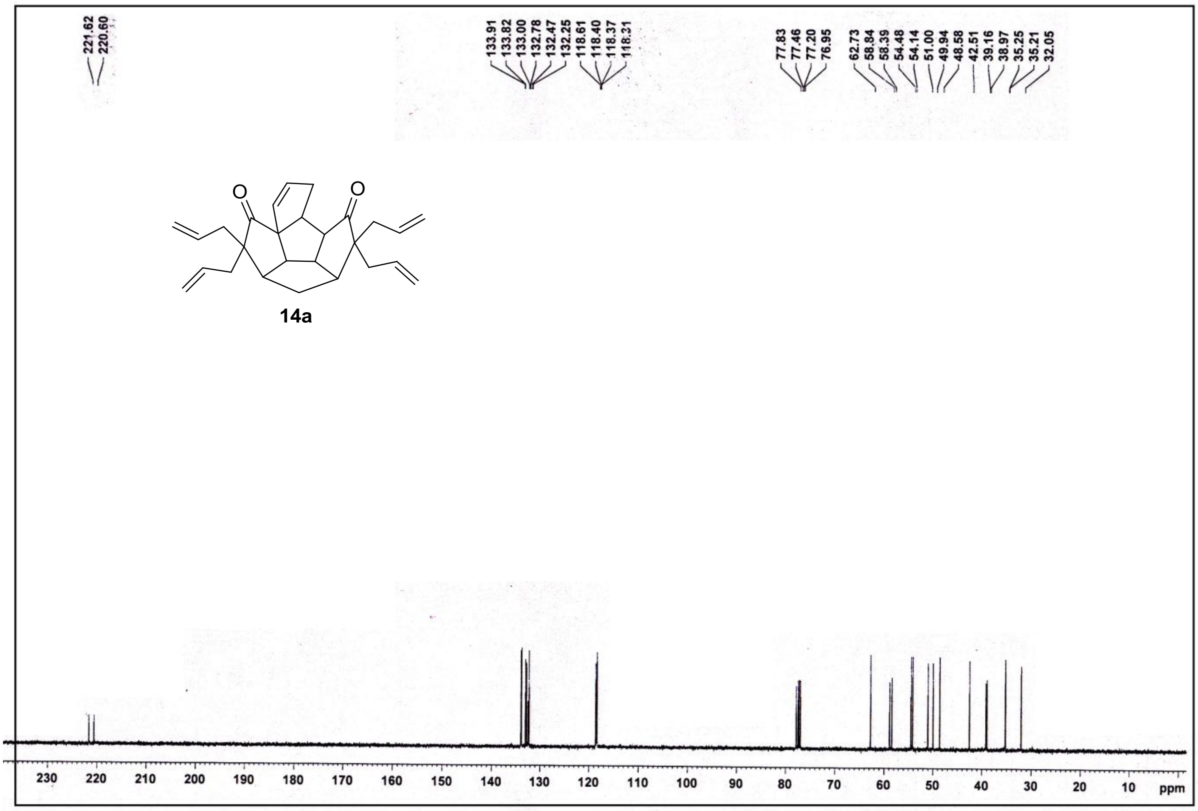

Compound 15a ^{1}H and ^{13}C NMR (400 MHz, $CDCl_3$)

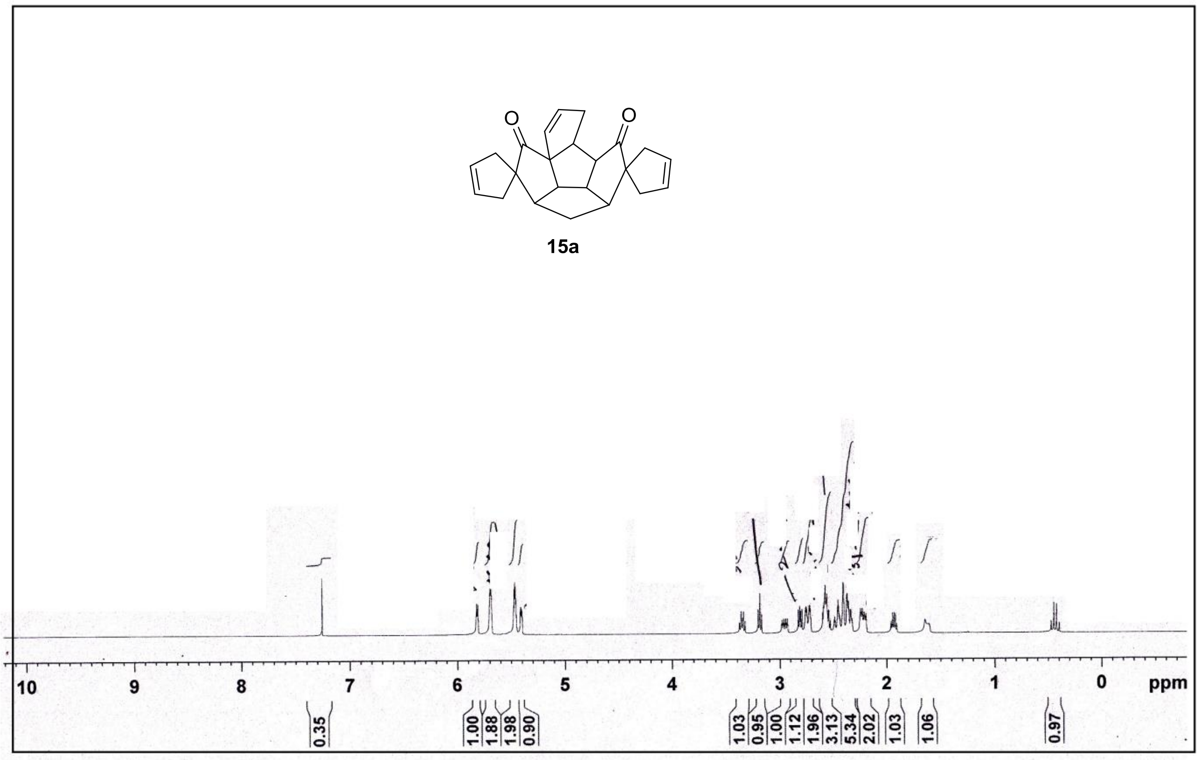

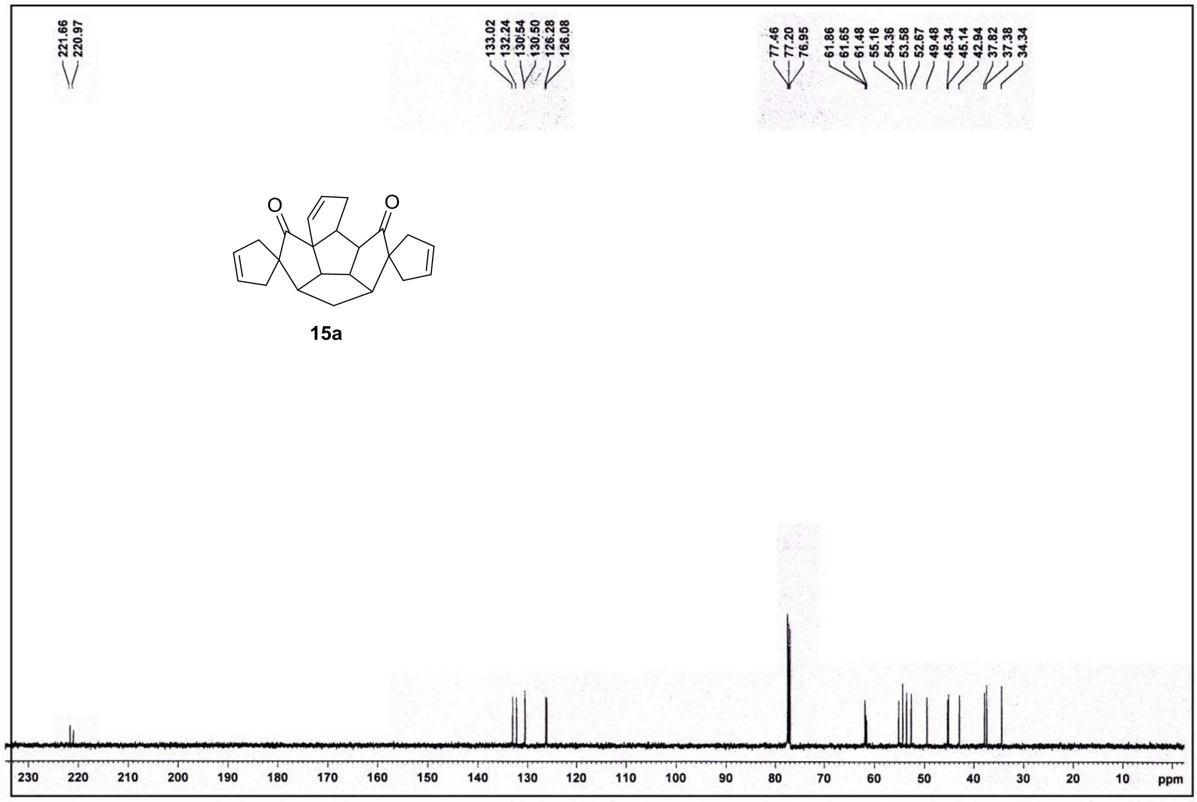

Compound 16a ^{1}H and ^{13}C NMR (500 MHz, $CDCl_3$)

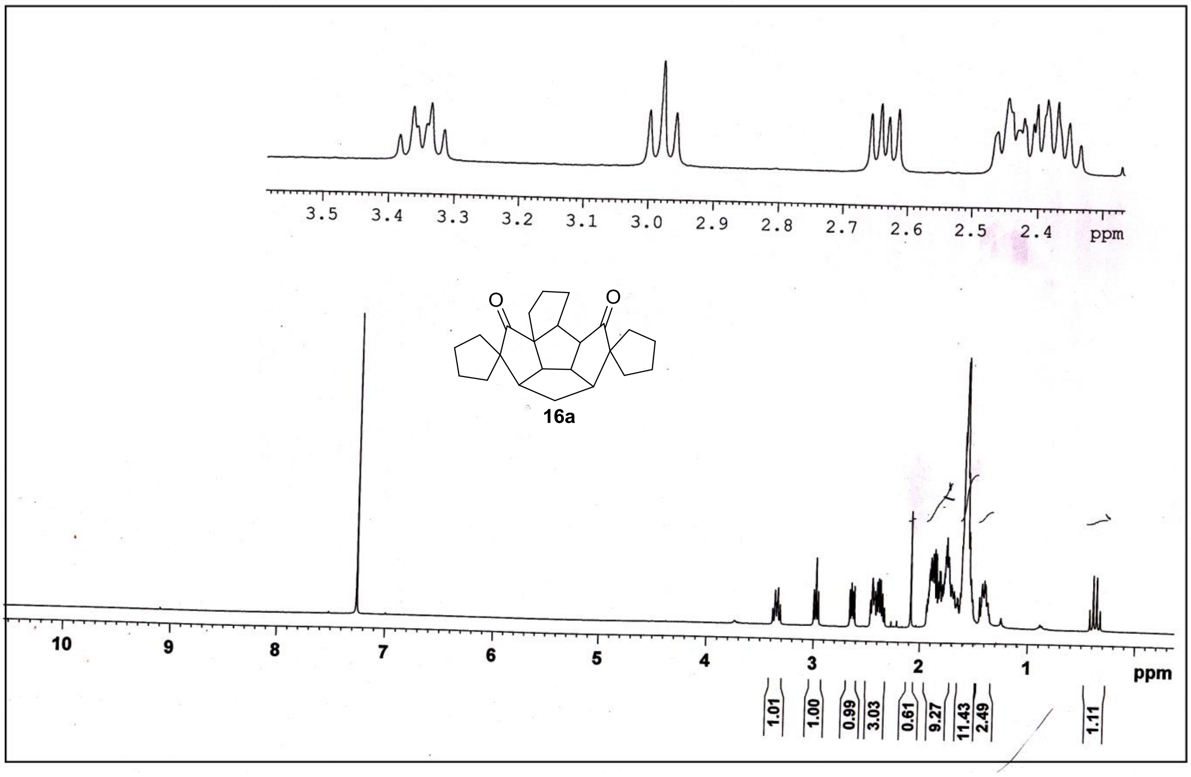

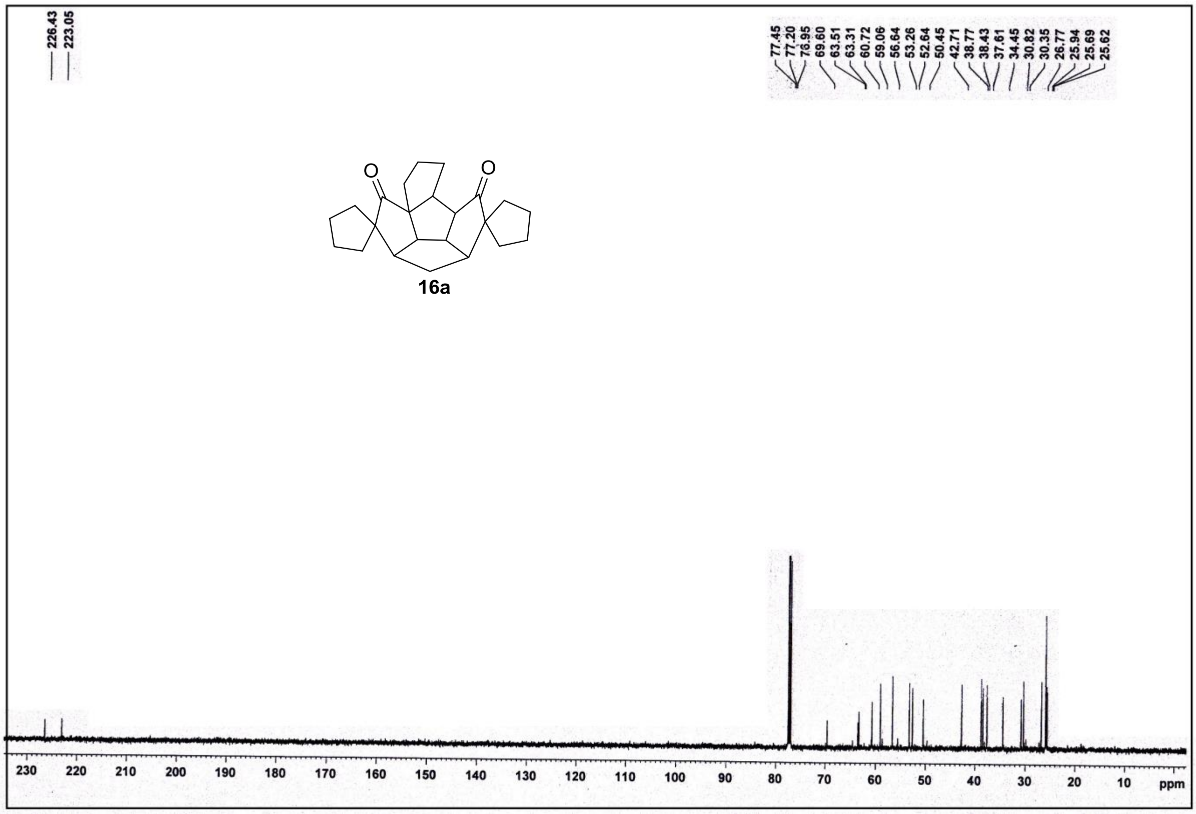

Compound 14b ^{1}H and ^{13}C NMR (400 MHz, CDCl$_3$)

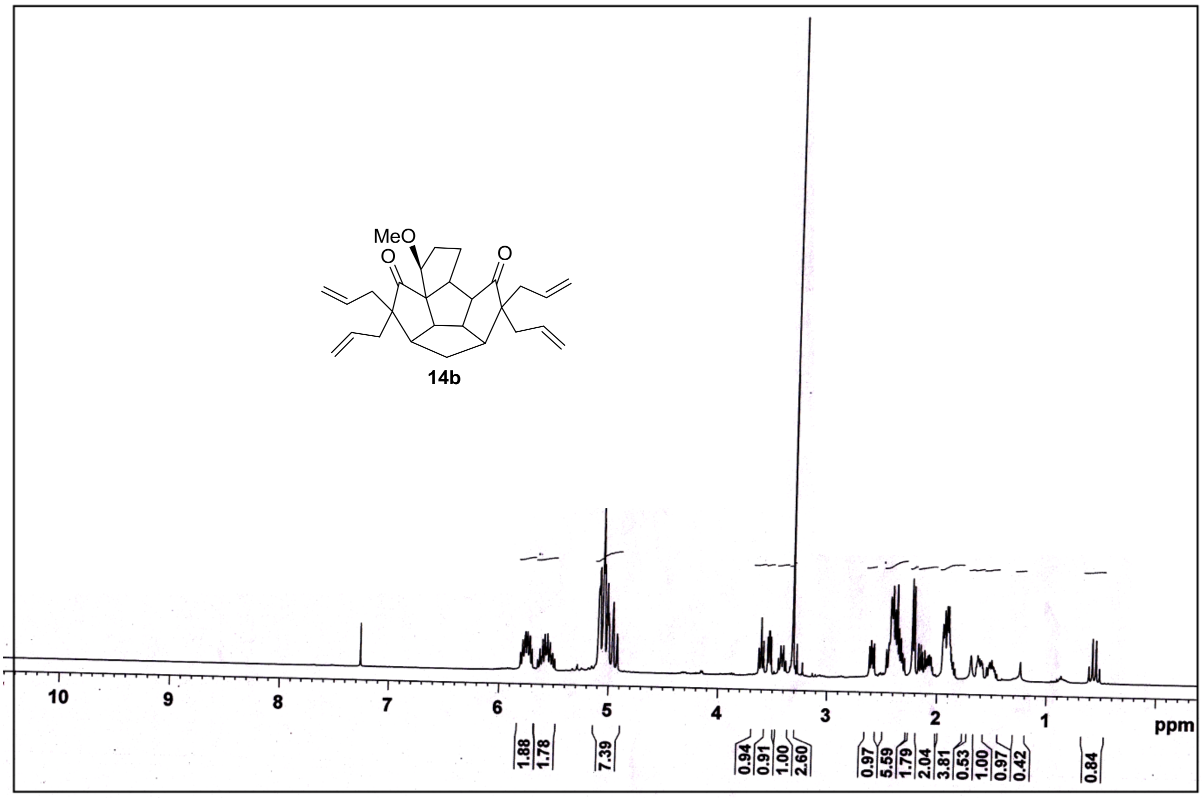

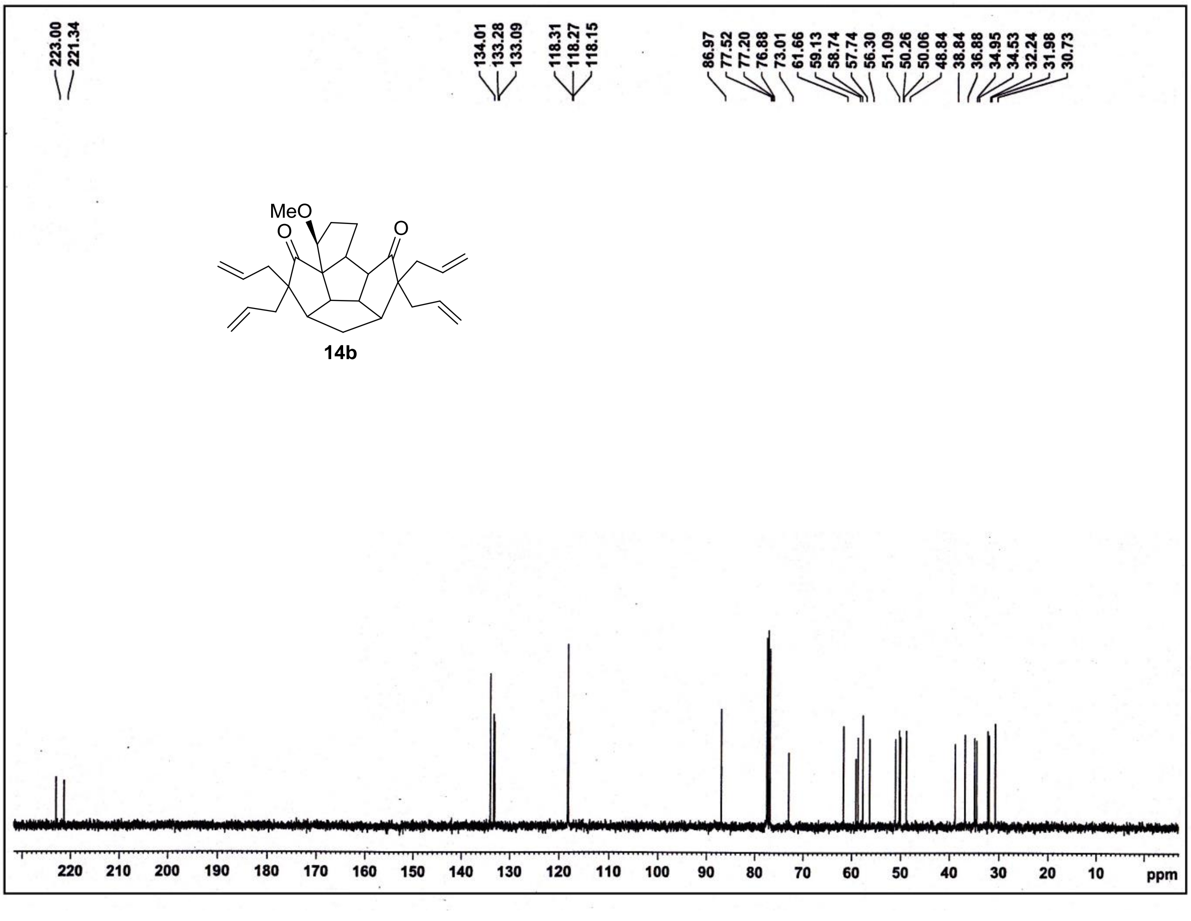

Compound 15b ^{1}H and ^{13}C NMR (500 MHz, $CDCl_3$)

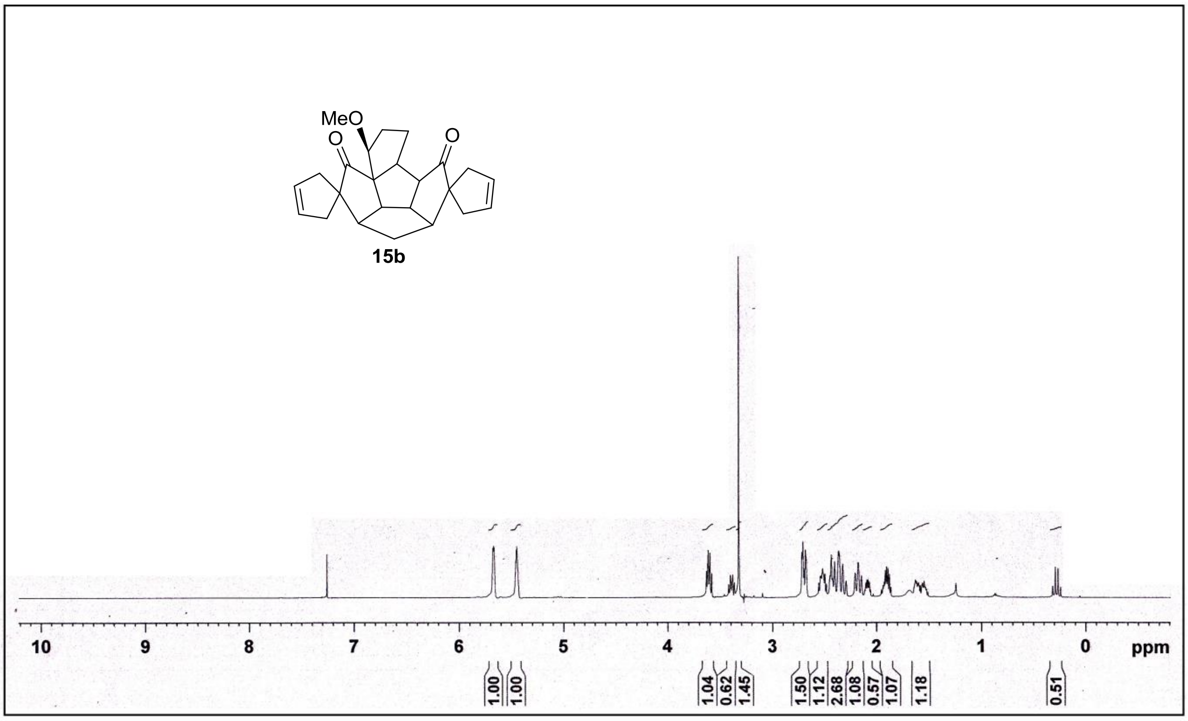

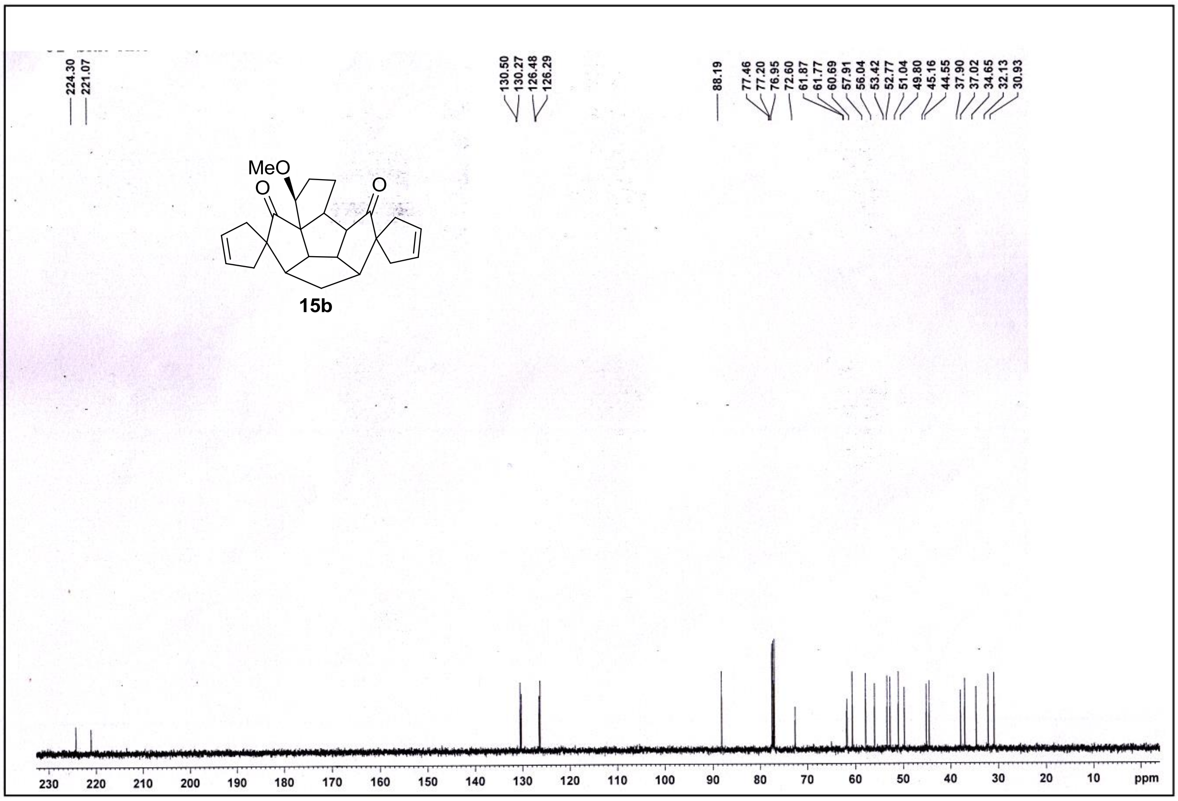

Compound 16b ^{1}H and ^{13}C NMR (400 MHz, $CDCl_3$)

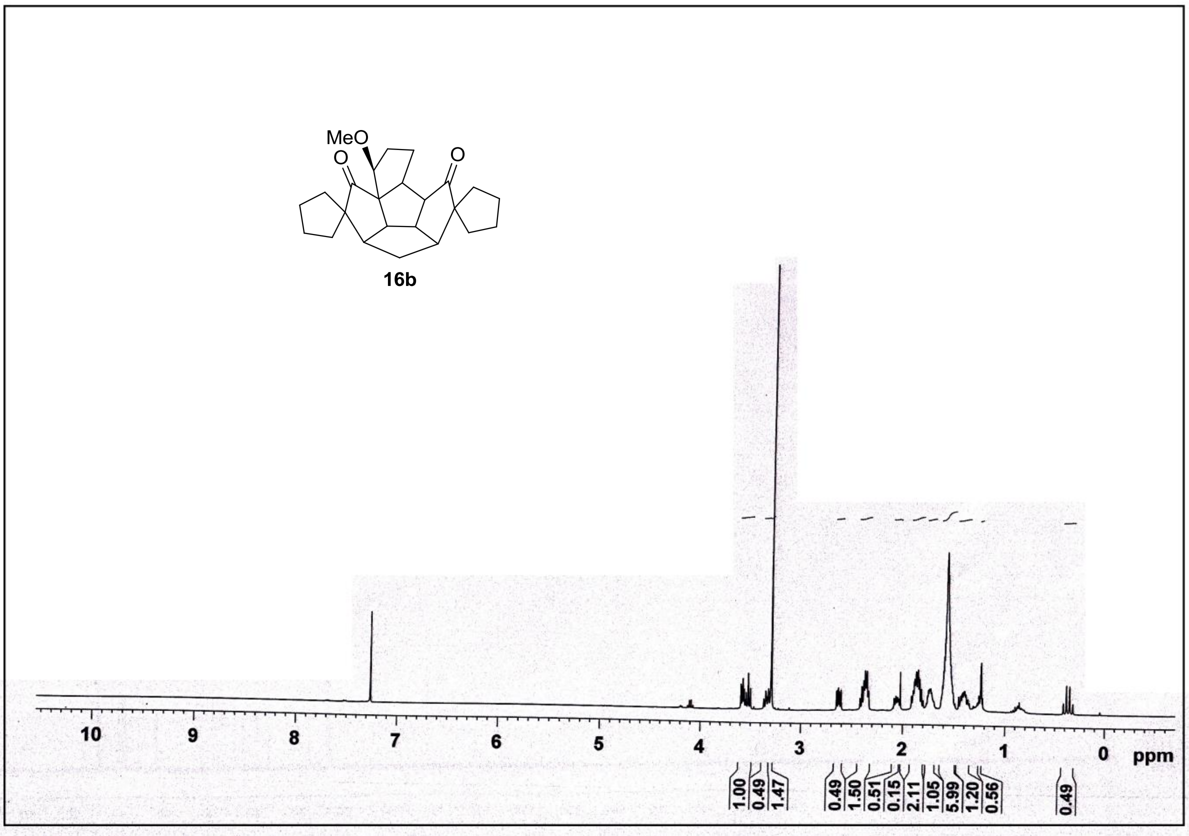

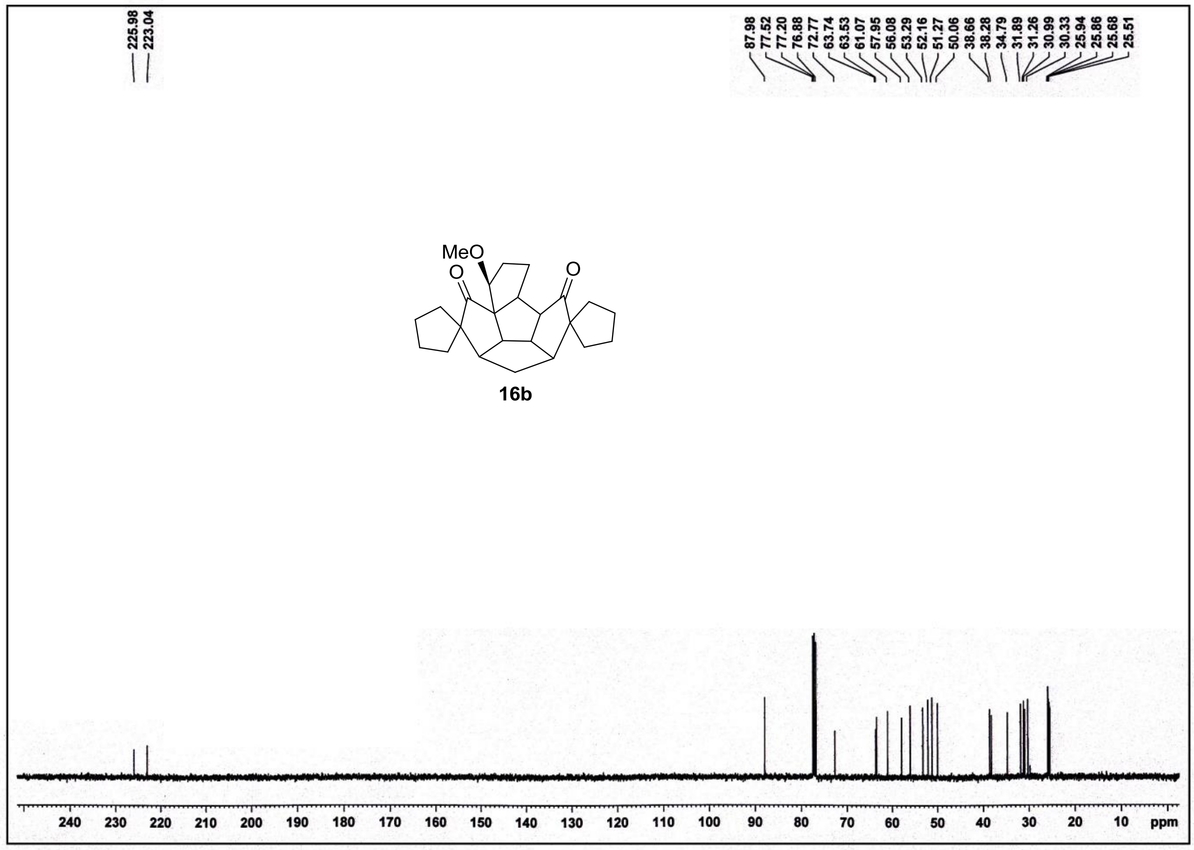

Compound 14c ^{1}H and ^{13}C NMR (400 MHz, $CDCl_3$)

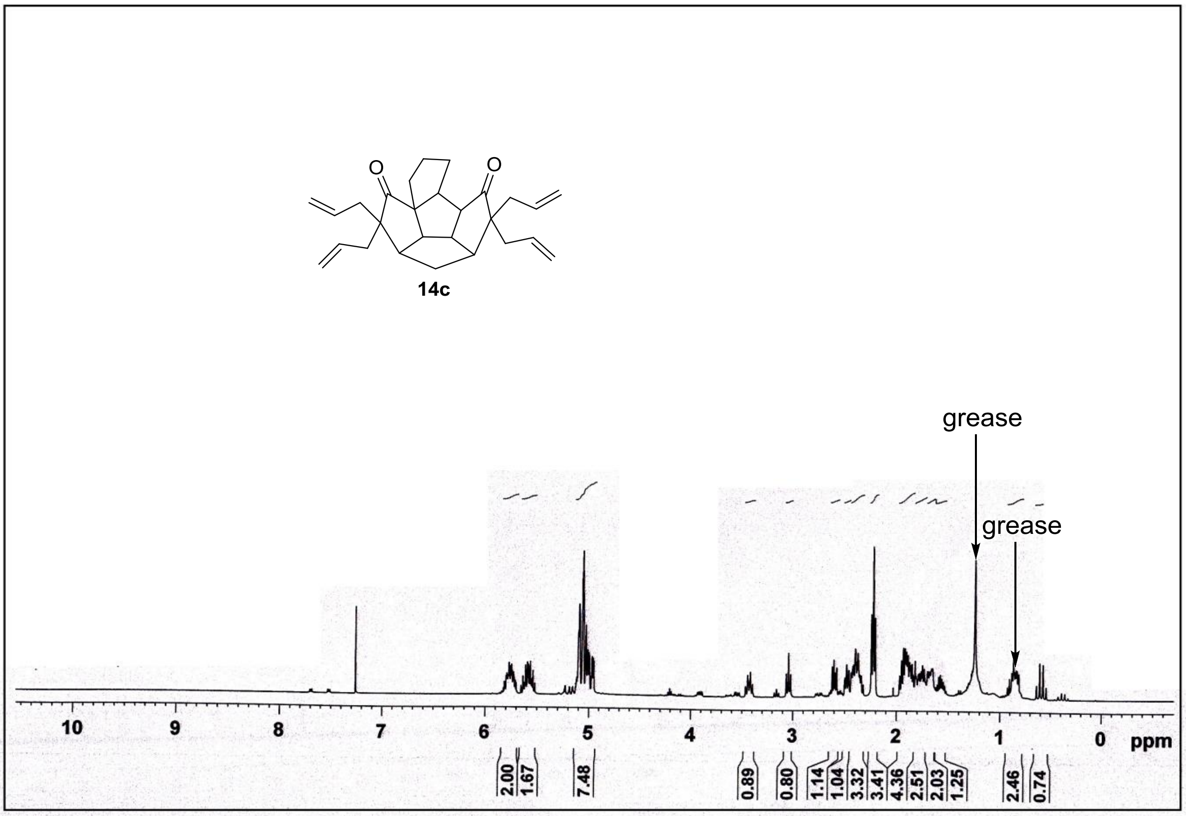

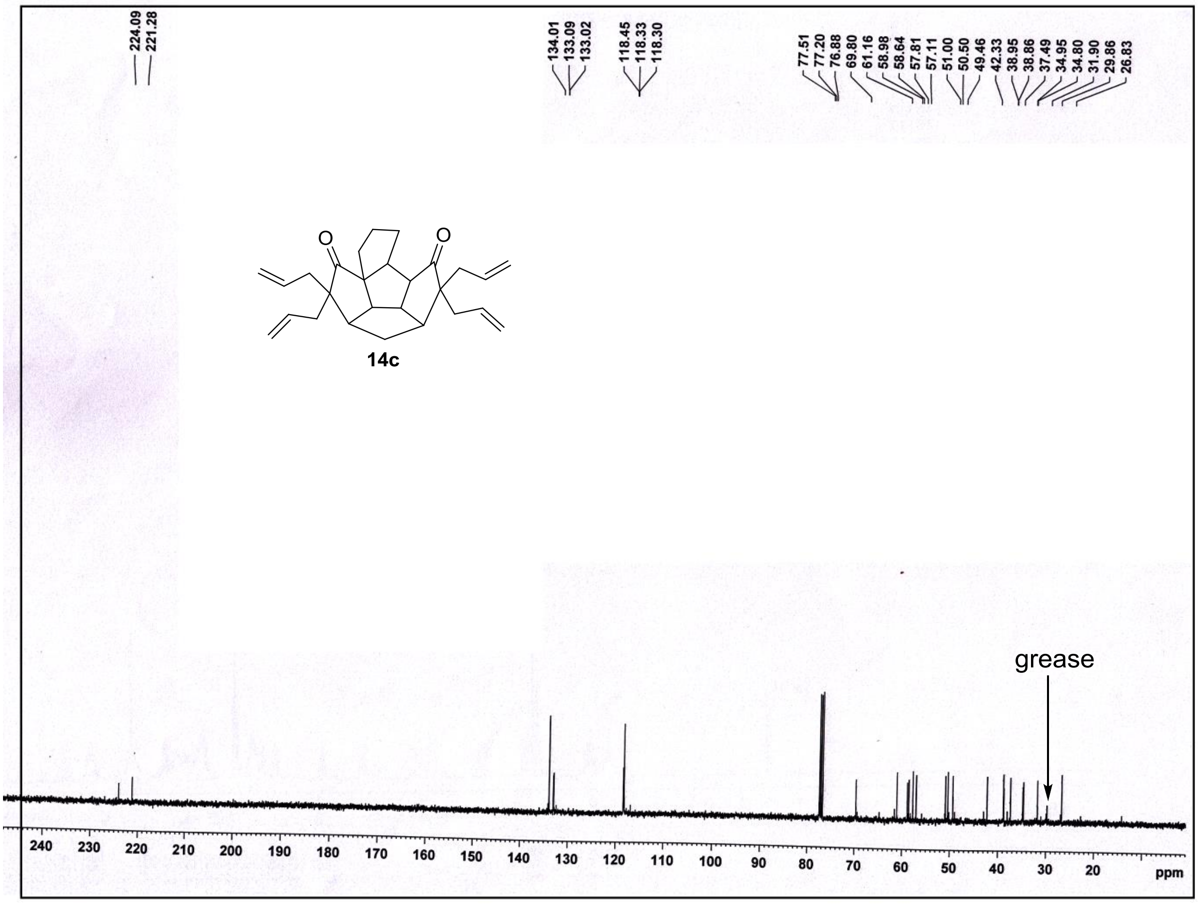

Compound 15c ^{1}H and ^{13}C NMR (500 MHz, $CDCl_3$)

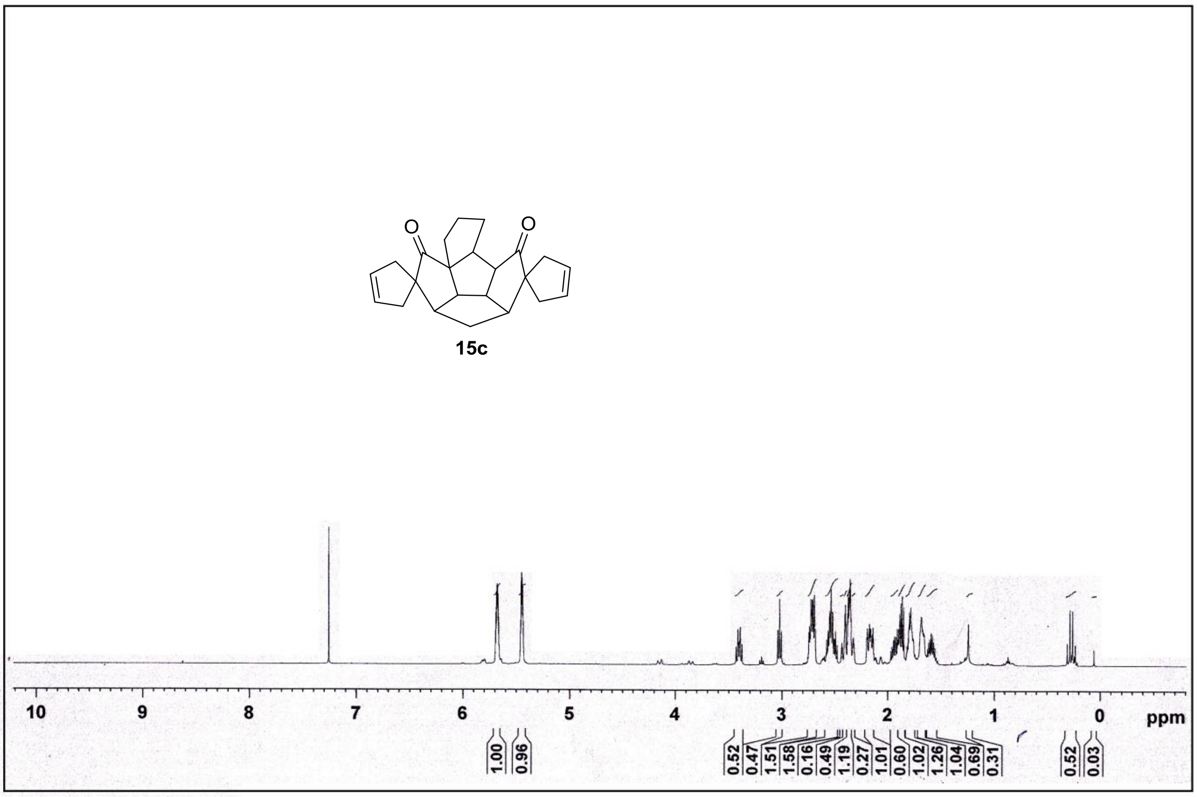

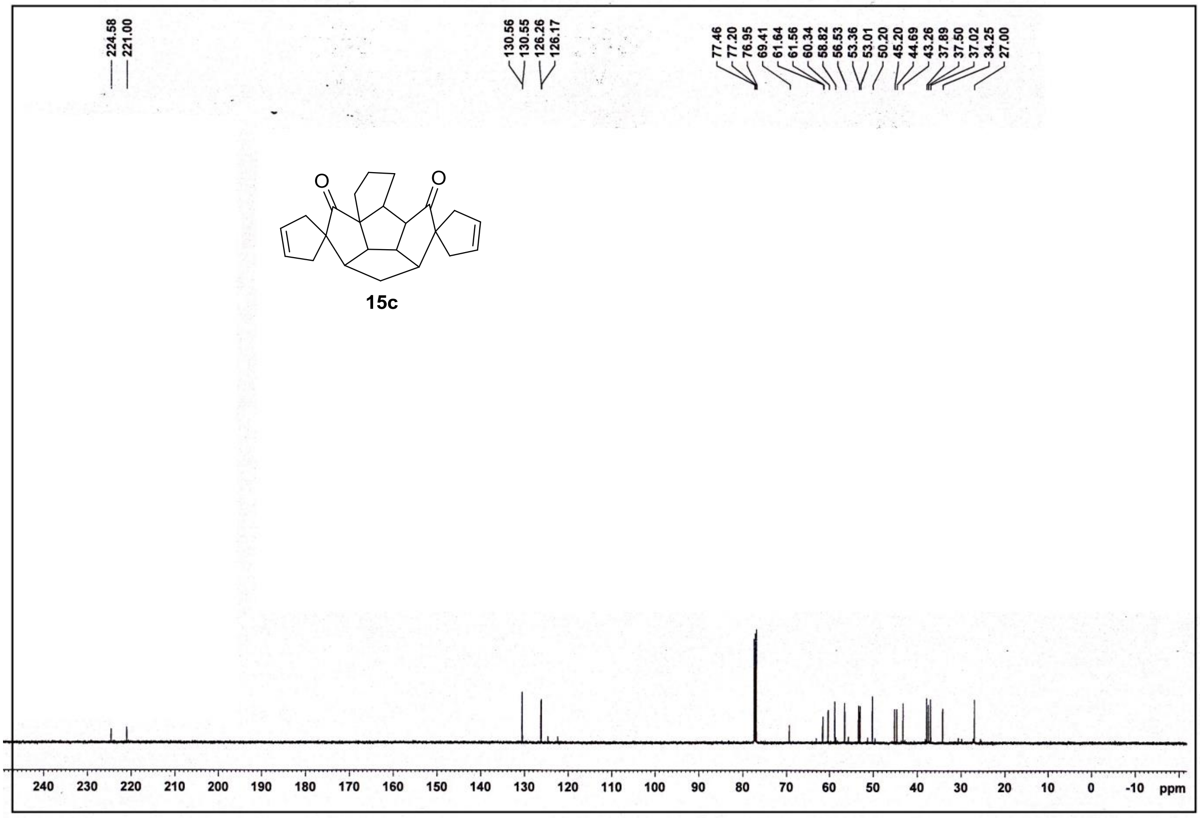

Compound 14d ^{1}H and ^{13}C NMR (500 MHz, CDCl$_3$)

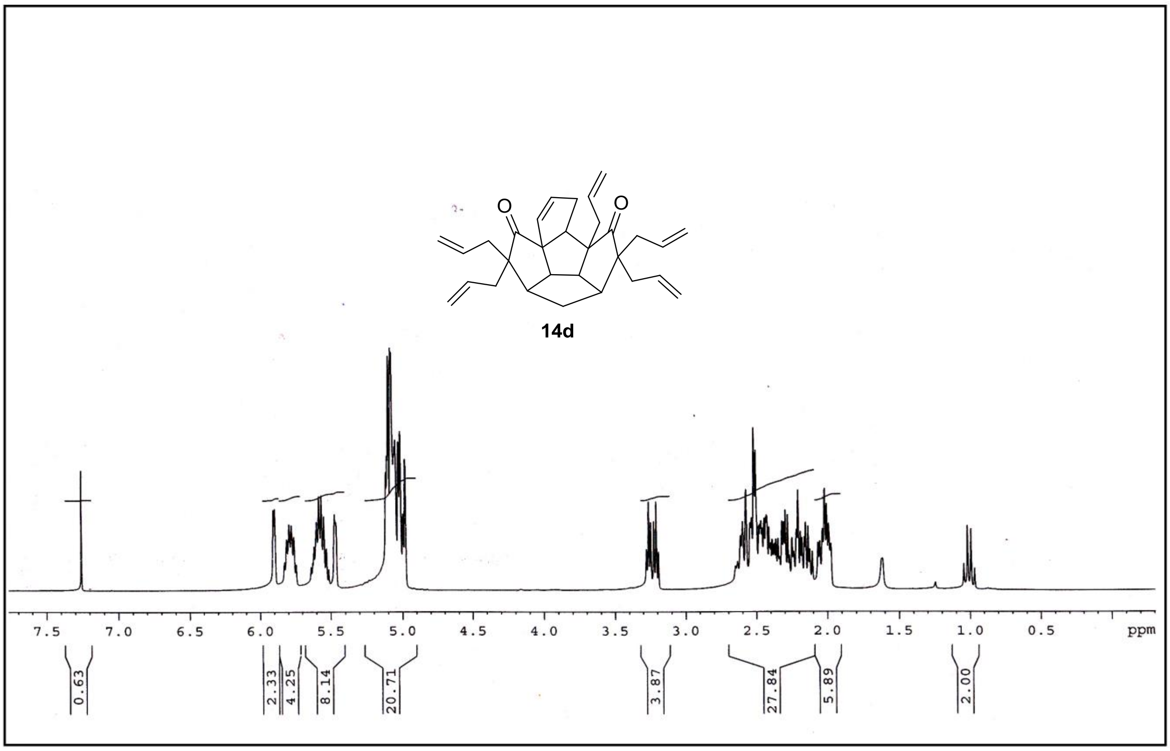

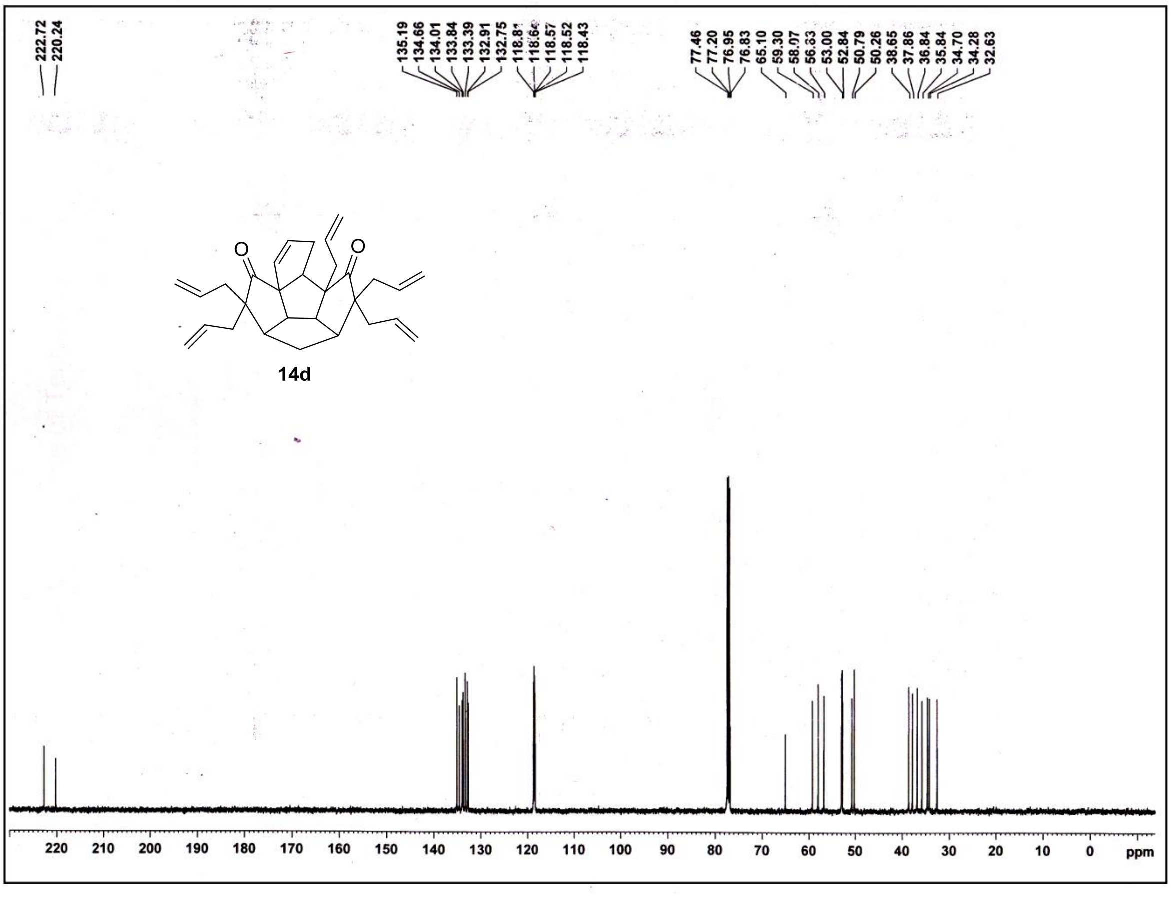

Compound 15d ^{1}H and ^{13}C NMR (500 MHz, $CDCl_3$)

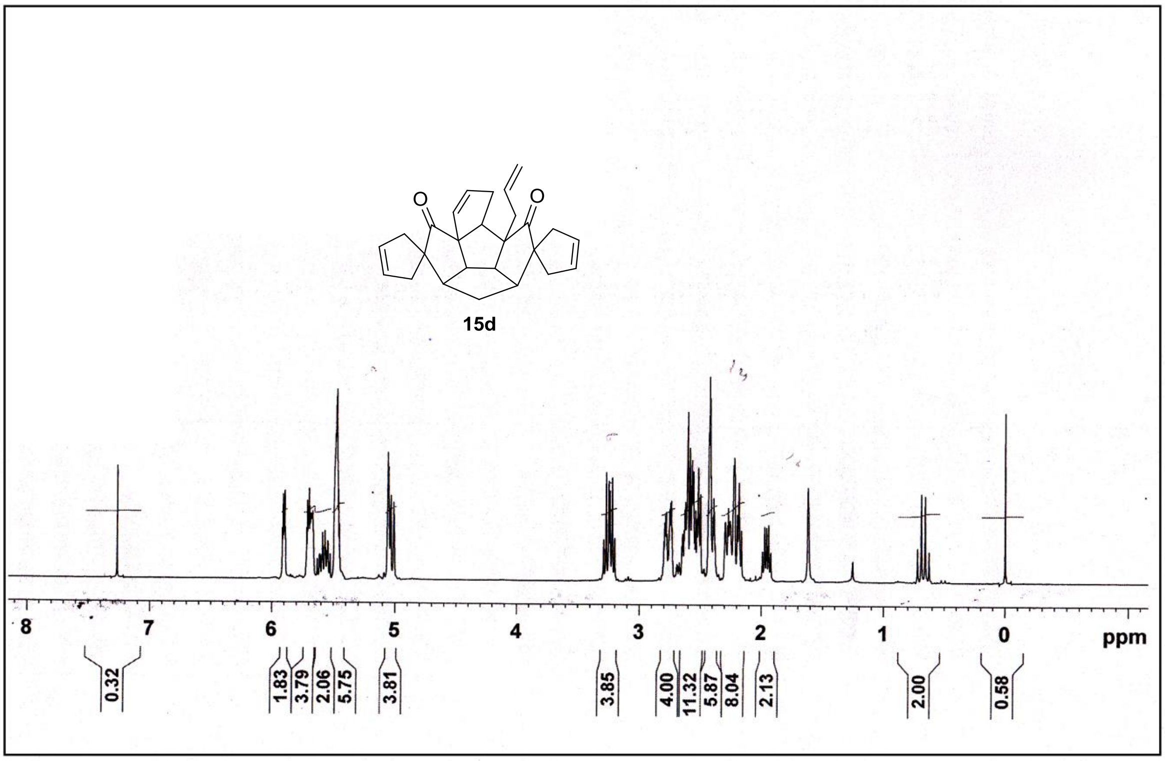

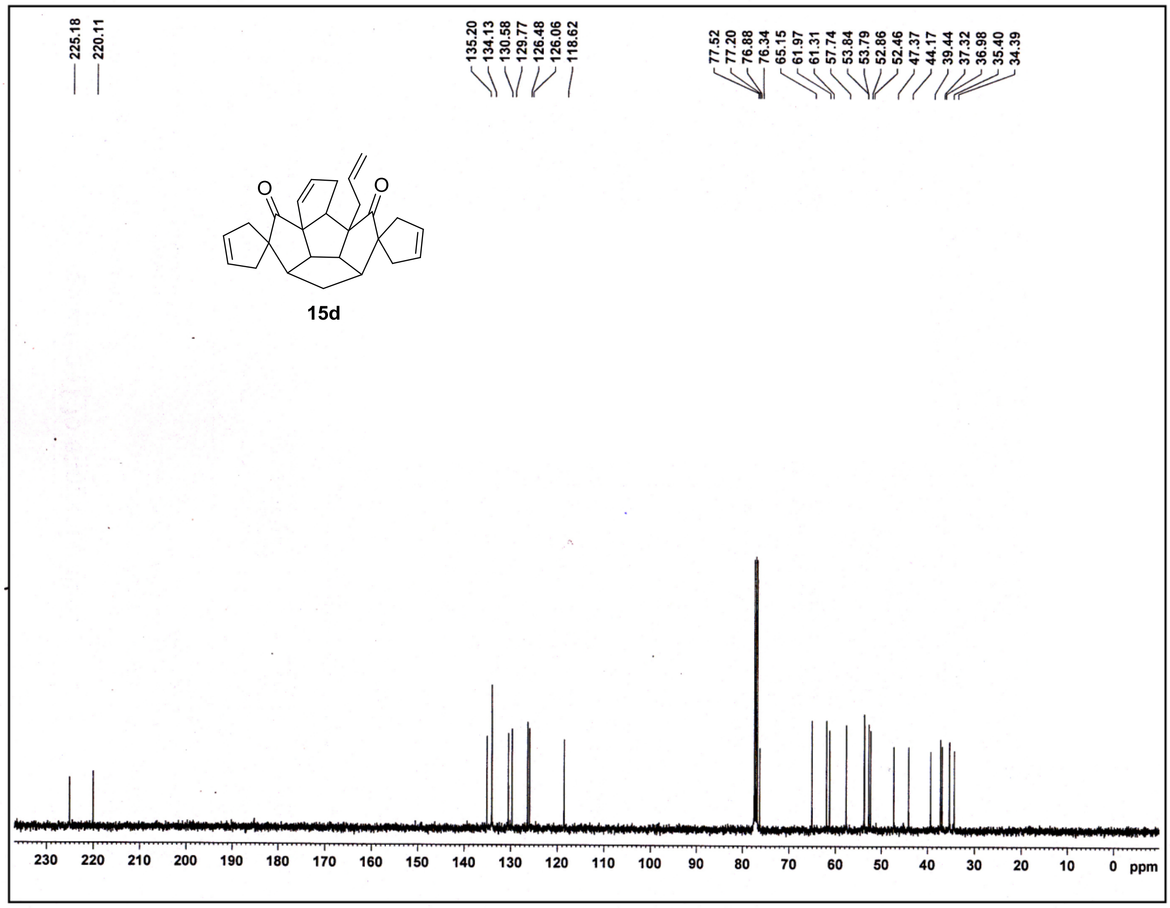

Compound 16d ^{1}H and ^{13}C NMR (500 MHz, $CDCl_3$)

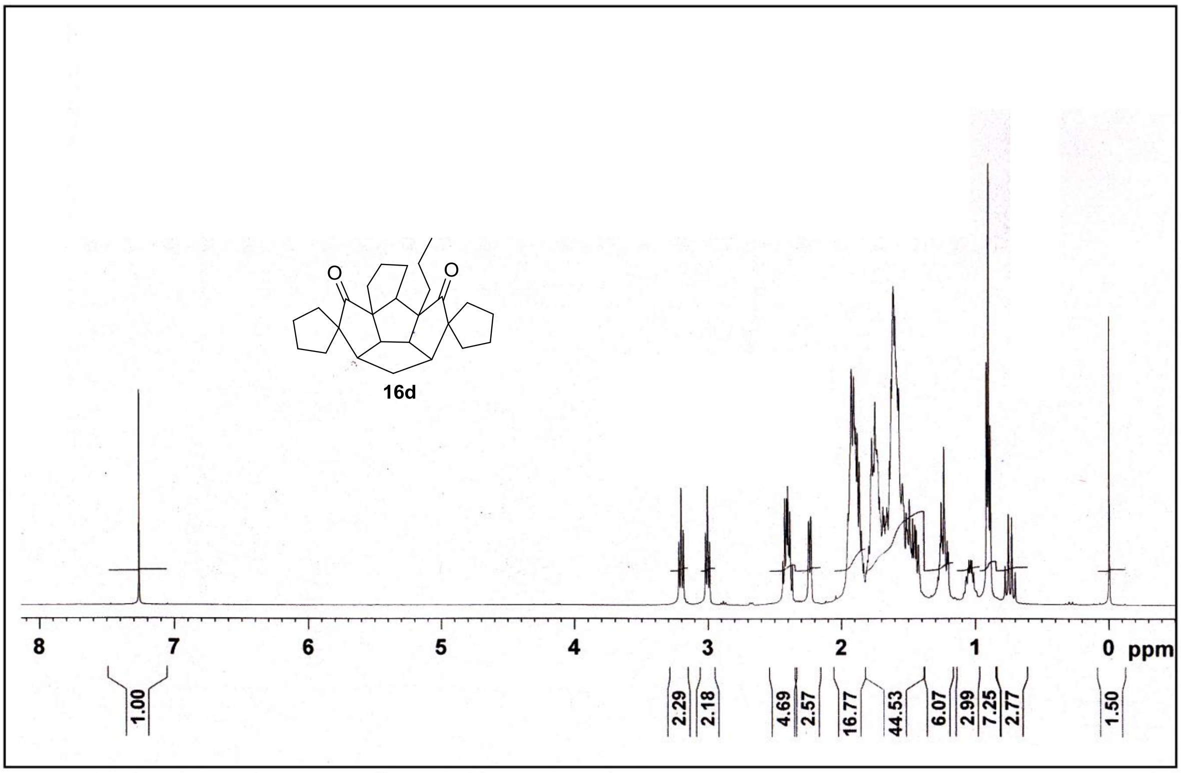

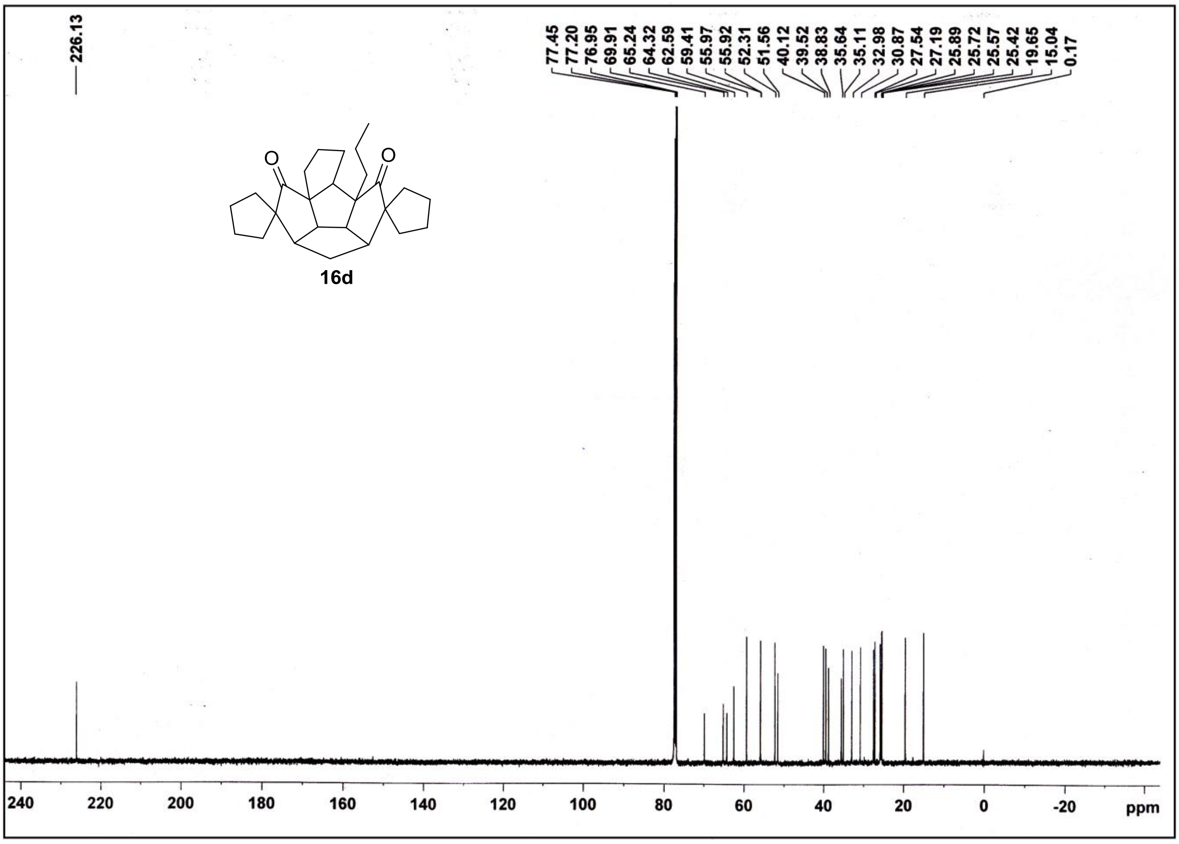

Supplement: File 2 — NMR spectra. [file Beilstein_J_Org_Chem-11-1123-s002.pdf]
